# Differential genetic mutations of ectoderm, mesoderm, and endoderm-derived tumors in TCGA database

**DOI:** 10.1186/s12935-020-01678-x

**Published:** 2020-12-11

**Authors:** Xingjie Gao, Xiaoteng Cui, Xinxin Zhang, Chunyan Zhao, Nan Zhang, Yan Zhao, Yuanyuan Ren, Chao Su, Lin Ge, Shaoyuan Wu, Jie Yang

**Affiliations:** 1 Department of Biochemistry and Molecular Biology, Department of Immunology, School of Basic Medical Sciences, Tianjin Medical University, Heping District Qixiangtai Road No.22, Tianjin, 300070 People’s Republic of China; 2 Key Laboratory of Immune Microenvironment and Disease, Ministry of Education, Key Laboratory of Cellular and Molecular Immunology in Tianjin, Excellent Talent Project, Tianjin Medical University, Tianjin, 300070 China; 3 Laboratory of Neuro-Oncology, Tianjin Neurological Institute, Department of Neurosurgery, Tianjin Medical University General Hospital and Key Laboratory of Neurotrauma, Variation, and Regeneration, Ministry of Education and Tianjin Municipal Government, Tianjin, 300052 China

**Keywords:** Ectoderm, Mesoderm, Endoderm, Tumor, TCGA, Genetic mutation

## Abstract

**Background:**

In terms of biological behavior, gene regulation, or signaling pathways, there is a certain similarity between tumorigenesis and embryonic development of humans. Three germ layer structure exhibits the distinct ability to form specific tissues and organs.

**Methods:**

The present study set out to investigate the genetic mutation characteristics of germ layer differentiation-related genes using the tumor cases of the cancer genome atlas (TCGA) database.

**Results:**

These tumor samples were divided into three groups, including the ectoderm, mesoderm, and endoderm. Children cases less than 9 years old accounted for a larger proportion for the cases in the ectoderm and mesoderm groups; whereas the middle-aged and elderly individuals (from 50 to 89 years old) were more susceptible to tumors of endoderm. There was a better prognosis for the cases of mesoderm, especially the male with the race of White, compared with the other groups. A missense mutation was frequently detected for the cases of ectoderm and endoderm, while deletion mutation was common for that of mesoderm. We could not identify the ectoderm, mesoderm, or endoderm-specific mutated genes or variants with high mutation frequency. However, there was a relatively higher mutation incidence of endoderm markers (*GATA6*, *FOXA2*, *GATA4*, *AFP*) in the endoderm group, compared with the groups of ectoderm and mesoderm. Additionally, four members (*SMO, GLI1*, *GLI2*, *GLI3*) within the Hedgehog signaling pathway genes showed a relatively higher mutation rate in the endoderm group than the other two groups.

**Conclusions:**

TCGA tumors of ectoderm, mesoderm, and endoderm groups exhibit the distinct subject distribution, survival status, and genomic alteration characteristics. The synergistic mutation effect of specific genes closely related to embryonic development may contribute to the tumorigenesis of tissues or organs derived from the specific germ layers. This study provides a novel reference for exploring the functional connection between embryogenesis and tumorigenesis.

## Background

It has commonly been assumed that the embryonic period occurred in the first 3 months of human intrauterine development [1]. After the combination of the spermatozoa in males with the ovum in females, the formation and the followed first mitotic division of the zygote cell indicate the beginning of a complicated embryonic development process [1, 2]. On the third day of gestation, it forms the morula structure upon the action of cleavage; then it will become hollow vesicles, which is called the “blastocyst” [2, 3]. The implantation of the blastocyst into the endometrium follows the formation of a bilaminar structure, including epiblast and hypoblast [2, 3]. Next, the three complete germ layers, including ectoderm (outer layer), mesoderm (middle layer), and endoderm (inner layer), are developed [2, 3].

A germ layer is a primary group of cells involved in the preliminary formation of external and internal body shape [3, 4]. In brief, the ectoderm structure eventually differentiates into the tissues or organs of the epidermis, sensory system, nervous system, gland; the mesoderm structure can form the urinary system, reproductive system, circulatory system, hematopoietic system, motor system, and connective tissue; the endoderm structure gives rise to the development of the respiratory epithelium, intestinal epithelium, digestive gland epithelium, and so on [2, 3, 5–9]. Different species show distinct embryonic morphological and developmental characteristics [1, 5, 10, 11]. A variety of genetic regulatory mechanisms underlying the germ layer determination and differentiation contribute to the transformation from the initial gamete fusion to various multicellular tissues or organs of the body [8, 9, 12, 13].

Interestingly, a series of issues (e.g., biological behavior, gene regulation, or signaling pathways, etc.) during embryogenesis show a certain similarity with that of tumorigenesis [14–16]. The web-based TCGA database systematically enrolls numerous cases with more than 30 types of tumors in different tissues or organs and contains the large-scale genome sequencing datasets [17, 18]. In the present study, we divided TCGA tumor samples into three groups of ectoderm, mesoderm, and endoderm, and explored the genomic alteration features of tumors from the perspective of embryonic development.

## Materials and methods

### Classification of three germ layer

According to the main process of three germ layer differentiation (Additional file 1: Fig. S1), the datasets of TCGA (https://cancergenome.nih.gov/) were divided into three groups, including the ectoderm, mesoderm, and endoderm. We presented the brief classification information of TCGA cases in Additional file 2: Fig. S2. The adrenal gland tumor of the TCGA database was classified into the groups of mesoderm and ectoderm, respectively, based on the terms of the cortex and medulla. In addition, we excluded the tumors with uncertain or controversial sources and included the bladder epithelial tumor in the endoderm group.

### Survival curve analysis

We got the datasets of the sample size, vital status, survival time, gender, and race from the TCGA databases. Then, a Kaplan–Meier overall survival curve analysis was performed by the IBM SPSS Statistics 20 Software. A log-rank test was applied to the comparison of survival status. When the *P* value was less than 0.05, we considered the statistical difference.

### Oncogrid analysis

Based on the “Oncogrid” module (https://portal.gdc.cancer.gov/exploration?searchTableTab=oncogrid) within the TCGA database, we visualized the information of the top 50 mutated genes for the top 500 tumor cases, including mutation type, mutation frequency, gender, race, ethnicity, age at diagnosis, vital status, and days to death, in the ectoderm, mesoderm and endoderm groups, respectively. Also, we provided the combined Oncogrid results of these groups.

### Germ-specific mutation site detection strategy

Based on the factors of clinical characteristics and sample size, we further classified each germ layer group into three subgroups. Then, these subgroups in each group were subjected to a Venn diagram analysis to obtain the commonly mutated genes or mutation sites, which then overlapped with the other two germ layer groups. Thus, the potential specific mutant genes/sites widely presented in the respective germ layer group were obtained. The “Launch Analysis” module of TCGA and on-line Venn tool (http://bioinformatics.psb.ugent.be/webtools/Venn/) were applied for the above Venn diagram analysis.

### Expression, mutation, and survival analysis of germ layer markers

We utilized the “Multiple Gene Comparison” module of gene expression profiling interactive analysis approach (version 2) [19] to profile the expression of three germ layer markers. Referencing the relevant publications [20–25], the ectoderm markers (*NES*, *TUBB3*, *SOX1*, *SALL3*), mesoderm markers (*MESP1*, *EOMES*, *TBXT*, *MIXL1*), and endoderm markers (*GATA6*, *FOXA2*, *GATA4*, *AFP*) were selected. The data was visualized by an interactive heatmap. Besides, we utilized the “Survival Analysis” module to perform the prognostic analyses of overall survival (OS) and disease-free survival (DFS). Furthermore, we used the approach of the cBio Cancer Genomics Portal [26, 27] to perform the mutation analysis of these markers. The results of the mutation spectrum and genetic alteration in the ectoderm, mesoderm, and endoderm groups were visualized by the “OncoPrint” module, respectively.

### Genetic mutation profile analysis of signaling pathways

Also, we performed the genetic mutation profile analysis of the embryogenesis-associated signaling pathways, including Hedgehog, Notch, TGFβ, and WNT pathways [5, 14, 28, 29], through the cBio Cancer Genomics Portal. The germ layer-associated members were analyzed: Hedgehog pathway (*PTCH1*, *SMO*, *SHH*, *GLI1*, *GLI2*, *GLI3*); Notch pathway (*NOTCH1*, *NOTCH2*, *NOTCH3*, *RBPJ*, *JAG1*, *HES5*); TGFβ pathway (*TGFB1*, *TGFB2*, *TGFB3*, *Nodal*, *BMP4*, *SMAD2*); Wnt pathway (*WNT1*, *WNT3A*, *WNT8A*, *CTNNB1*, *AXIN2*, *FGF4*).

## Results

### Classification and characteristics of TCGA tumor cases

**Fig. 1:**
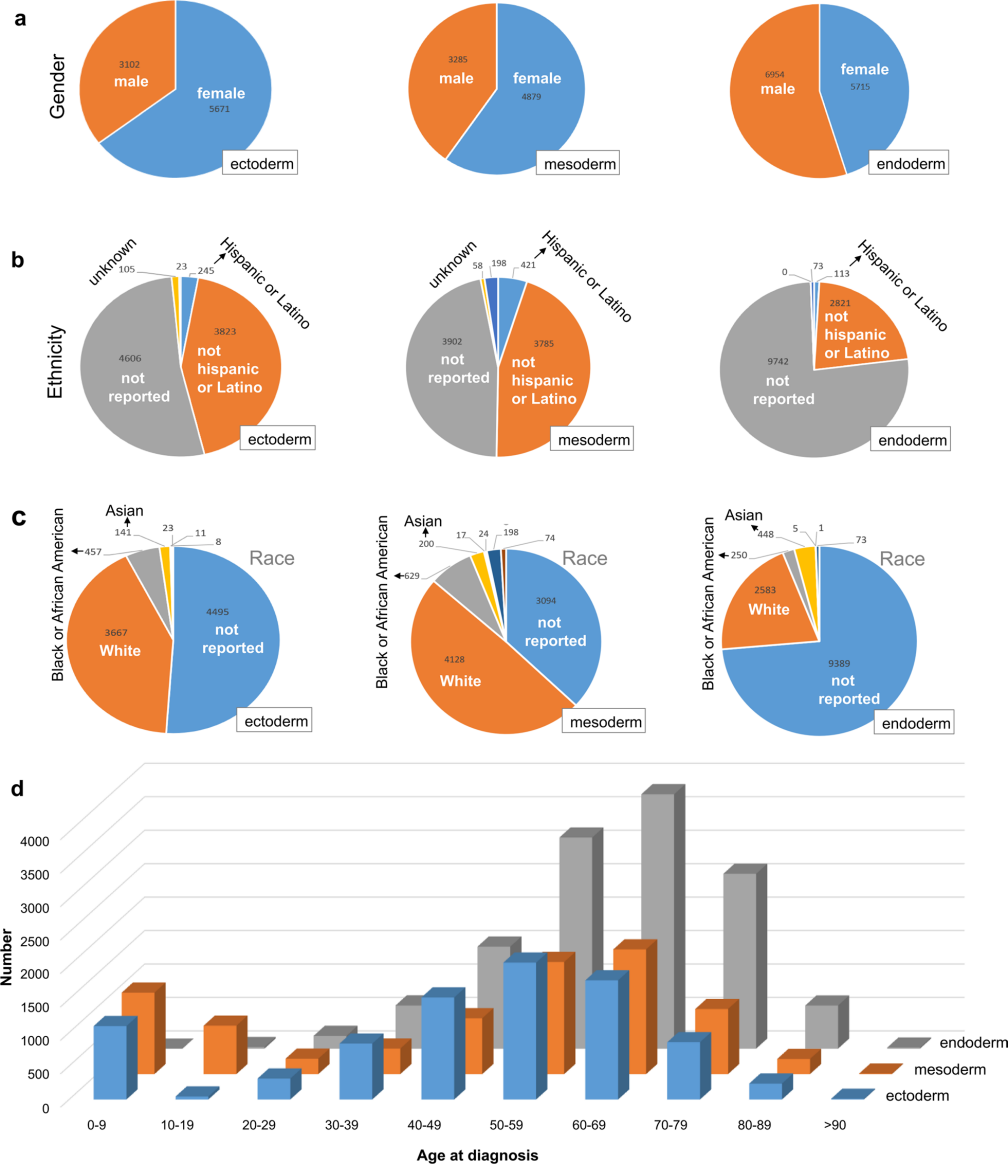
Distribution characteristics of TCGA tumor cases. **a** Gender; **b** Ethnicity; **c** Race; **d** Age at diagnosis

**Fig. 2:**
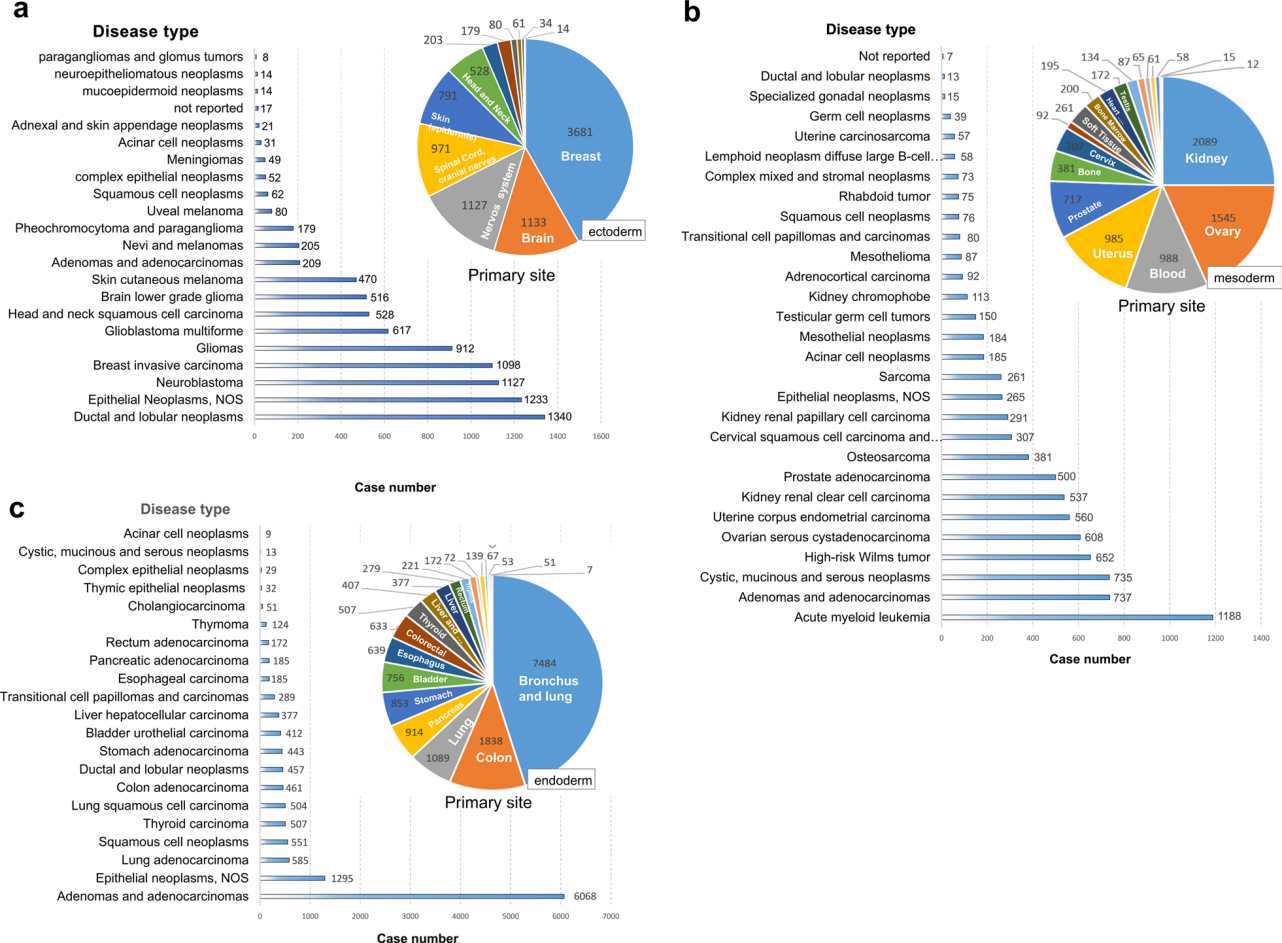
Disease type information of TCGA tumor individual. **a** Ectoderm; **b** mesoderm; **c** endoderm

We first divided the tumor cases of TCGA database into three groups, including ectoderm, mesoderm, and endoderm. Then, the distribution characteristics of gender, ethnicity, race, and age at diagnosis of tumor cases were analyzed, respectively. As shown in Fig. 1a, female patients account for the most cases with the ectoderm or mesoderm-derived tumors, whereas a high percentage of male tumor cases was found in the endoderm group. Apart from the cases with the unavailable data, the tumor cases with the non-Hispanic or Latino ethnicity (Fig. 1b) and the white race (Fig. 1c) occupied a large proportion in these groups. For the distribution characteristics of age at diagnosis (Fig. 1d), we found that, compared with the endoderm group, the children cases (less than 9 years old) account for a larger proportion in the groups of ectoderm and mesoderm. However, the middle-aged and elderly cases (50 ~ 89 years old) were more prone to endometrial-derived tumor diseases. Regarding the tumor types, most cases in the ectoderm group suffered from the ductal and lobular neoplasms, epithelial neoplasms, neuroblastoma, and breast invasive carcinoma (Fig. 2a). The mesoderm group showed the highest proportion of “acute myeloid leukemia” type (Fig. 2b). But the “adenomas and adenocarcinomas” were the most common type in the endoderm group (Fig. 2c).

### Survival curve analysis data

**Fig. 3:**
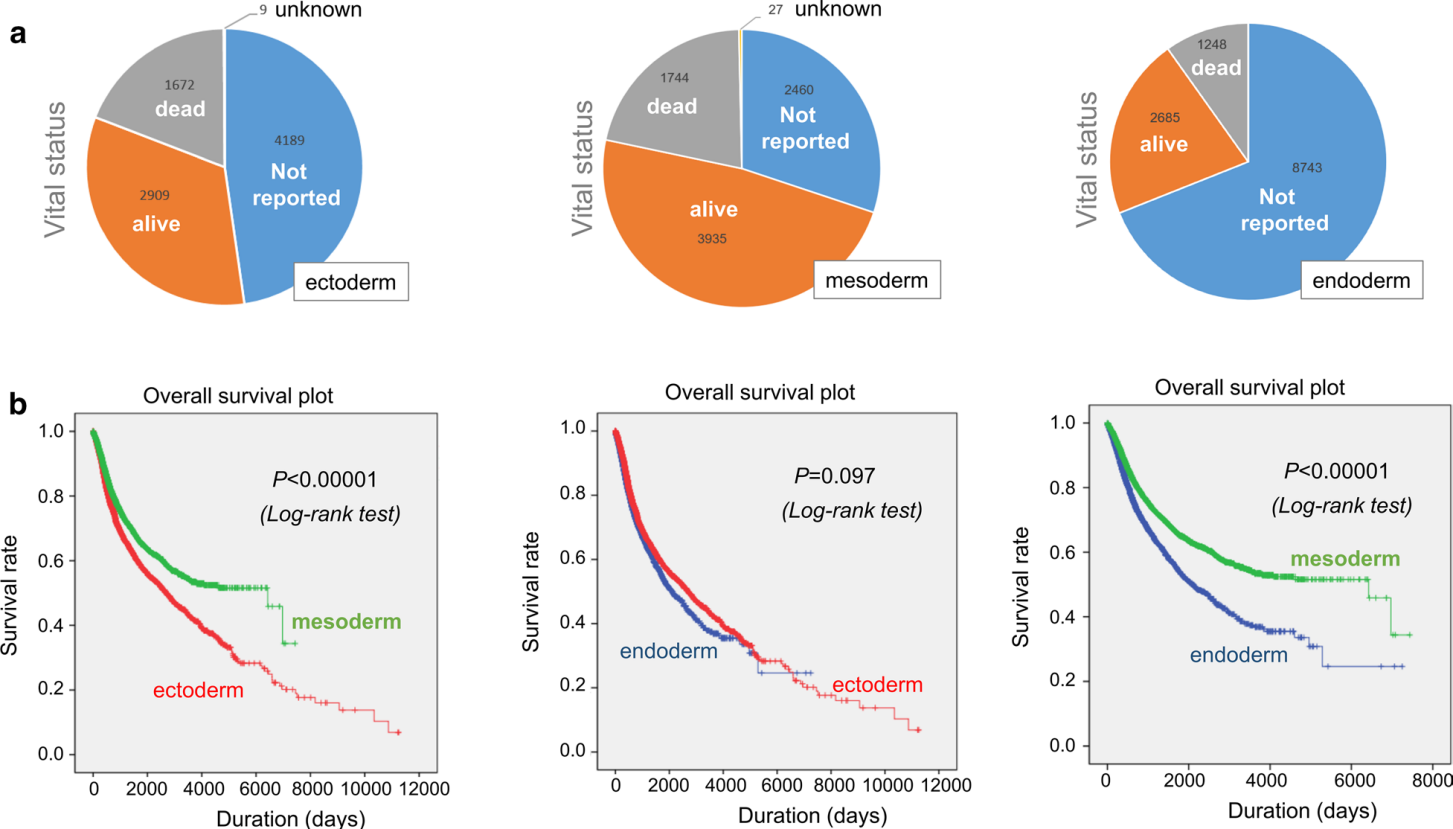
Survival curve analysis. **a** Vital status; **b** Overall survival plot data

Next, we performed the survival curve and log-rank analyses in the groups of ectoderm, mesoderm, and endoderm, respectively. As shown in Fig. 3a, all three groups contained the cases without the reported survival information and showed a higher proportion of alive status than dead status. The overall survival analysis data (Fig. 3b) showed that a better prognosis of cases in the mesodermal group, compared with that in the groups of endoderm or ectoderm (*P *< 0.00001). Nevertheless, there was no significant difference between the ectodermal and endoderm group in the clinical prognosis of cases (*P *= 0.097).

Based on the two factors (race and gender), we further performed a series of subgroup analyses of survival status. There was no significant difference among the three groups in the clinical prognosis of Asian cases (Additional file 3: Fig. S3a, *P* = 0.472). Nevertheless, we observed a better prognosis for the tumor cases with the race of White (Additional file 3: Fig. S3b, *P *< 0.001) and Black or African American (Additional file 3: Fig. S3c, *P *= 0.011) in the mesoderm group, compared with that in the endoderm or ectoderm group. Additionally, similar results were observed for the all male cases (Additional file 3: Fig. S3d, *P *< 0.001) or specific male cases with the white race (Additional file 3: Fig. S3e, *P *< 0.001), but not the female cases (Additional file 3: Fig. S3d, *P *= 0.086).

### Mutated gene analysis data

**Fig. 4:**
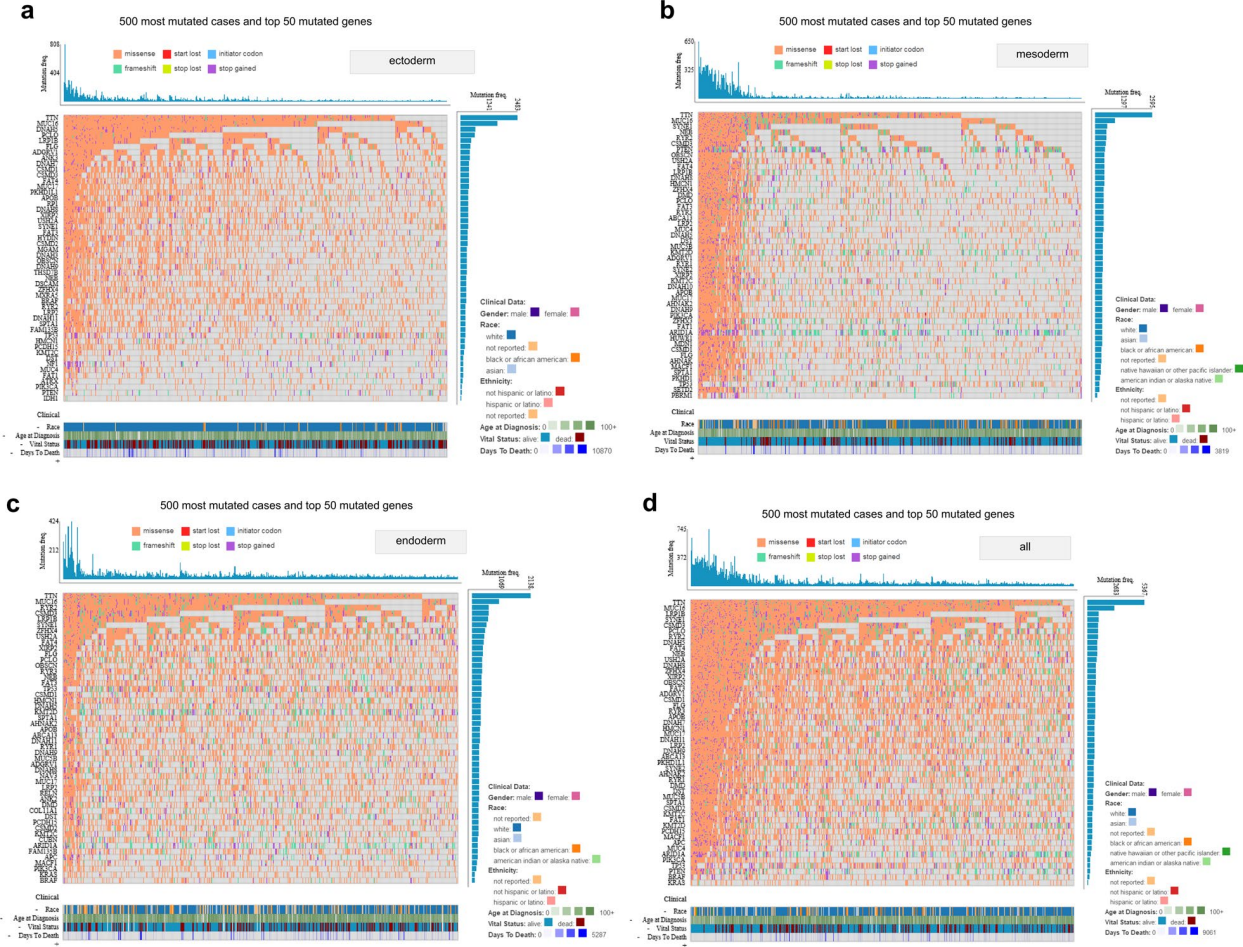
Oncogrid analysis using top 50 mutated genes in 500 most mutated cases. **a** Ectoderm; **b** mesoderm; **c** endoderm; **d** Combined data

To further analyze the differences among the three groups in the genetic mutations, we used the “Oncrgrid” module of TCGA to display the clinical information of the top 500 tumor cases with the top 50 mutated genes, including mutation type, mutation frequency, gender, race, ethnicity, age at diagnosis, vital status, and days to death. As shown in Fig. 4, the mutated gene number and mutation frequency in the mesoderm group were higher than that in the ectoderm and endoderm groups. When comparing to the ectoderm group, there was a higher rate of a frameshift mutation in the groups of mesoderm and endoderm (Fig. 4, relatively more green dots for frameshift).

Also, we are interested in the investigation of the genetic mutation status of mRNAs, lincRNAs, and miRNAs among the three germ layer source groups. As shown in Additional file 4: Fig. S4a-c, based on the mutation rate, we analyzed and displayed the top ten genes with the high mutant frequency in the ectoderm, mesoderm, and endoderm groups, respectively. There were some commonly mutated genes (Additional file 4: Fig. S4a-c, marked by red dots), such as *TP53*, *TTN*, *MUC16* (mRNAs); *XIST*, *TSIX*, *RP11*-*85G18.6* (lincRNAs); and *MIR1270*, *AC215219.2*, *AC090825. 1* (miRNAs), among these three groups. Then, we performed an intersection analysis of three sets of mutant genes by a Venn diagram. Additional file 4: Fig. S4d presented the list of the top ten mutated genes with relative specificity for the three germ layer source groups. Further, we analyzed the mutated gene-associated disease types. As shown in Additional file 4: Fig. S4e, the relatively specific lincRNA *RP11*-*65L19.4* gene mutation in the ectoderm group occurred only in four breast cancer cases and two skin cancer cases; *PPP1R2P9 AP00345.1* and *C16orf95* genes in the mesoderm group mainly existed in the cases with uterus tumors. Similarly, only seven cases, including three bladder cancer cases, three lung cancer cases, and one esophagus cancer case, comprised the *SNORA71E* gene mutation in the ectodermal group (Additional file 4: Fig. S4e). Therefore, we sensed that the identified specific mutated genes may not occur widely and frequently in each germ layer source group.

### Specific mutation site analysis data

**Fig. 5:**
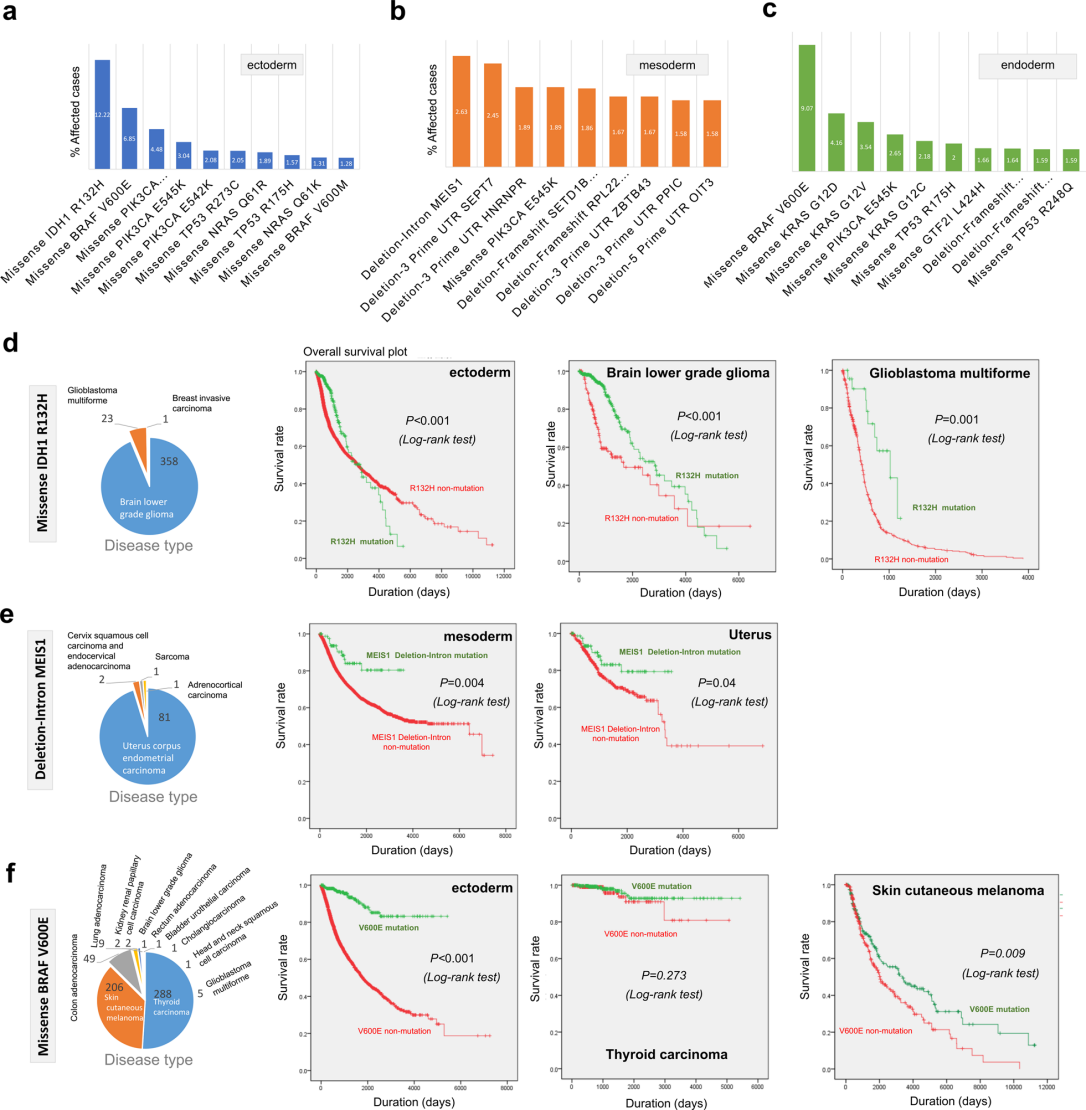
Analysis of mutated gene sites. **a**–**c** Top ten mutated gene sites in the ectoderm, mesoderm, and endoderm groups; **d** The disease type and overall survival plot analysis of missense *IDH1* R132H mutation; **e** The disease type and overall survival plot analysis of missense deletion-intron *MEIS1* mutation; **f** The disease type and overall survival plot analysis of missense *BRAF* V600E mutation

We should note that the above-stated mutant gene refers to a gene with one or more mutation sites. To focus on the characteristics of a specific genetic mutation site, we determined the mutation sites with high frequency in the ectoderm, mesoderm, and endoderm groups, respectively. Figure 5a–c lists the top ten genetic mutation sites with a higher frequency of occurrence. The missense mutations were frequently detected in the ectoderm and endoderm groups, whereas the deletion mutations were common in the mesoderm group. Then, we analyzed the R132H site of the *IDH1* gene, which showed the highest rate of incidence, in the ectoderm group, and found that this point mutation mainly occurred in the glioma cases, including 358 cases of brain lower-grade glioma (LGG) and 23 cases of glioblastoma multiforme (GBM) (Fig. 5d). The survival prognosis of tumor or glioma patients with R132H mutation was better than the cases with R132H non-mutation of the *IDH1* gene (Fig. 5d, *P* < 0.001). Besides, the *MEIS1* deletion mutation with the highest incidence in the mesoderm group occurred mainly in 81 patients with uterus corpus endometrial carcinoma. The followed survival analysis data also showed a better prognosis for the patients in the *MEIS1* deletion mutation group, compared with the *MEIS1* deletion non-mutation group (Fig. 5e, *P* < 0.05). In the ectoderm group, The *BRAF* V600E missense mutation with the highest incidence was mainly presented in 288 cases with thyroid carcinoma and 206 cases with skin cutaneous melanoma (Fig. 5f). Also, we observed that the tumor cases with *BRAF* V600E mutation of ectoderm showed a better survival prognosis than that with the V600E non-mutation (Fig. 5f, *P *< 0.001). However, a similar result was detected for the skin cutaneous melanoma (Fig. 5f, *P *= 0.009), but not thyroid carcinoma (*P *= 0.273).

Next, we tried to identify the common genetic mutation sites among three germ layer groups. Due to the limitation of pooled sample sizes, the first 50,000 gene loci of each group were set for a Venn diagram analysis. As shown in Additional file 5: Fig. S5a, 47,185 relatively specific sites were identified in the ectoderm group, 45,574 sites were in the mesoderm group, and 45,576 sites were in the endoderm group. Additional file 5: Fig. S5b-d lists the top ten gene loci with high incidence in each group, respectively. *NRAS* Q61K missense mutation in the ectodermal group mainly occurs in the patients with skin cancer, while splice acceptor *GATA3* X309_splice mutations only present in the breast cancer cases derived from ectoderm (Additional file 5: Fig. S5b). Multiple deletion mutations in the mesoderm group (e.g., Deletion-3 prime UTR *ADAR*, *BHLHE40, ZFX*, etc.) mainly occur in the cases with uterine tumors (Additional file 5: Fig. S5c). The top ten relative sites of the endoderm group were mostly frameshift mutations, and the first two mutation sites mostly occurred in the cases with digestive tract tumors (Additional file 5: Fig. S5d).

**Fig. 6:**
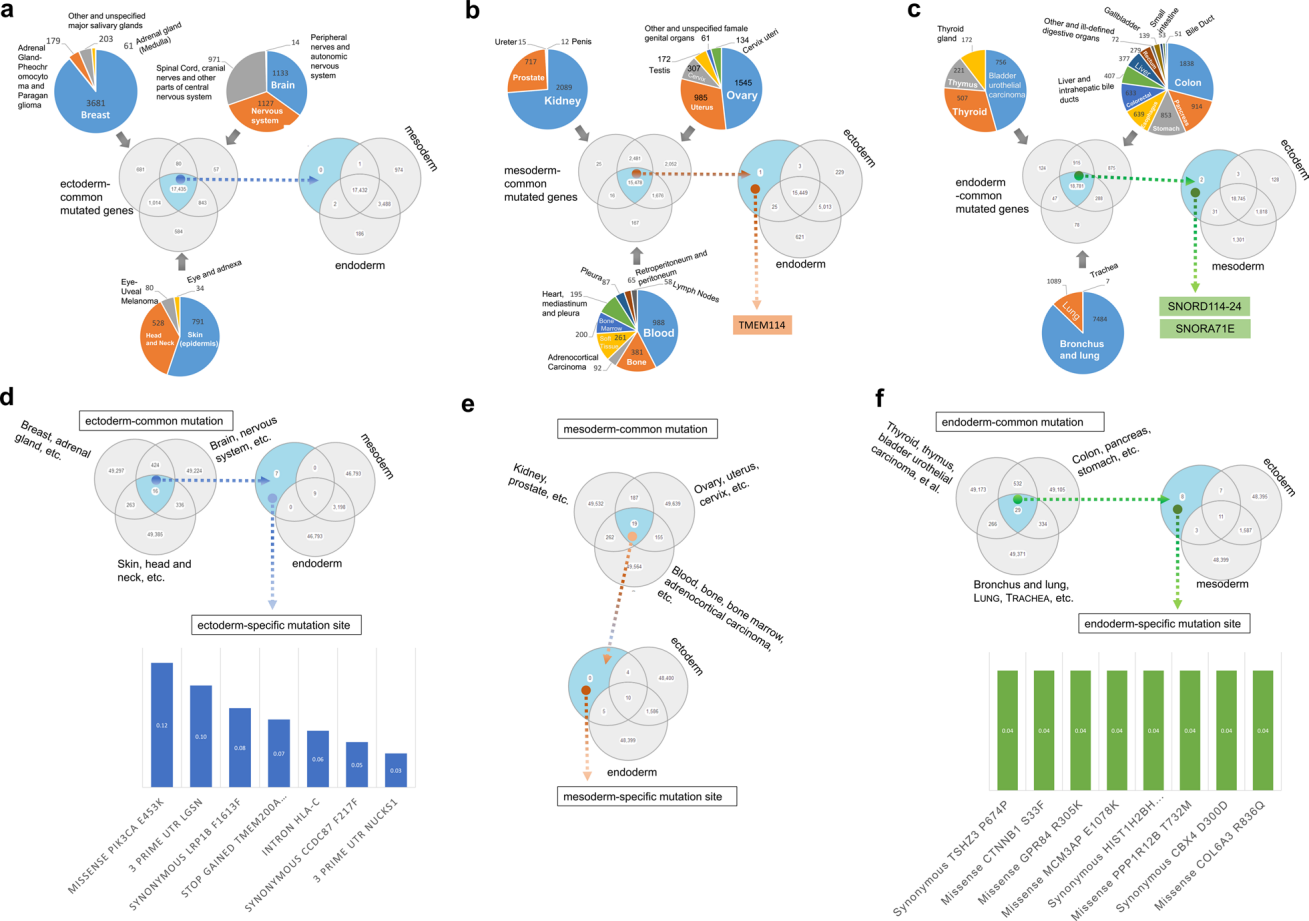
Analysis of ectoderm, mesoderm, and endoderm-specific and common mutated genes and sites. **a** The ectoderm-specific and common mutated genes analysis; **b** The mesoderm-specific and commonly mutated genes analysis; **c** The endoderm-specific and common mutated genes analysis; **d** The ectoderm-specific and common mutated site analysis; **e** The mesoderm-specific and common mutated site analysis; **f** The endoderm-specific and common mutated site analysis

We sensed that the above-mentioned gene locus mainly existed in less than two types of tumors. Thus, we tried to identify the possible germ layer-specific mutation sites. As shown in Fig. 6a, the ectoderm group was divided into three subgroups: (1) breast, adrenal gland, and salivary gland tumors; (2) brain and nervous system tumors; (3) skin and other tumors. After a Venn diagram analysis, 17,435 common mutant genes were obtained and crossed with all the mutated genes in the mesoderm and endoderm groups. Finally, no mutated gene unique to the ectoderm group was identified. In addition, we observed only one mesodermal group-specific mutant gene (*TMEM114*, Fig. 6b) and two endoderm-specific mutated genes (*SNORD114*-*24* and *SNORA71E*, Fig. 6c), which showed the very low frequency.

Next, we further utilized the same analysis strategy to obtain seven ectoderm group-specific gene mutation sites (Fig. 6d), eight endoderm group-specific mutation sites (Fig. 6f). However, there was no specific gene site was identified for the mesoderm group (Fig. 6e), There was also the question of low mutation frequency for these mutation sites. Our findings demonstrated that there exists no germ layer-specific gene mutation site with high frequency, which can be widely presented in most of the tumors within each germ layer group.

**Fig. 7:**
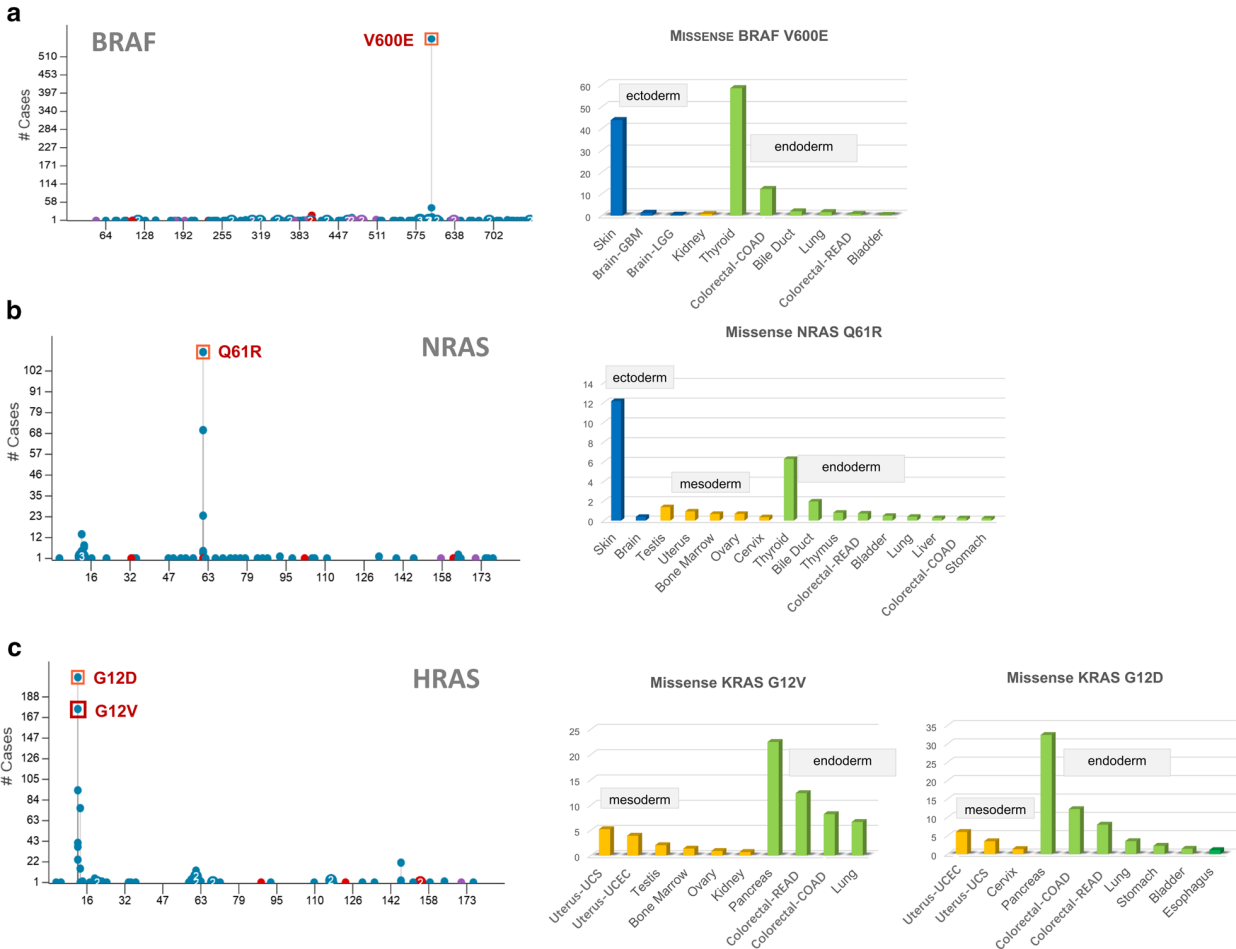
Distribution analysis of *BRAF* V600E, *NRAS* Q61R, and *HRAS* G12D/G12V. **a** *BRAF* V600E; **b** *NRAS* Q61R; **c** *HRAS* G12D/G12V

Considering the question of low mutation frequency, we analyzed the distribution features of some gene mutation sites with a high detection rate in the groups of ectoderm, mesoderm, and endoderm. The V600E mutation site of the *BRAF* gene (Fig. 7a) and the Q61R mutation site of the *NRAS* gene (Fig. 7b) were highly prevalent in ectoderm-derived skin cancer and endoderm-derived thyroid tumors. The G12V site and G12D of the *HRAS* gene (Fig. 7c) showed a high detection rate in endoderm-derived tumors.

Besides, there were mainly three gene mutation sites within the *PIK3CA* gene, namely E545K, E542K, and H1047R. E545K and E542K sites exhibited the highest detection rate in mesoderm-derived cervix tumors, whereas H1047R site mutation mainly occurred in ectoderm-derived breast cancer patients (Additional file 6: Fig. S6a). Within the *TP53* gene, the R175H site exhibited a high mutation rate in the endoderm-derived digestive tract tumors; R273C was more prevalent in the ectoderm-derived brain cancer, and R248Q was highly frequent in mesoderm-derived uterus tumors (Additional file 6: Fig. S6b). There was a main R132H mutation site within the *IDH1* gene, which was predominantly presented in the ectoderm-derived brain lower-grade glioma (Additional file 6: Fig. S6c).

### Mutation and expression analysis of germ layer markers

**Fig. 8:**
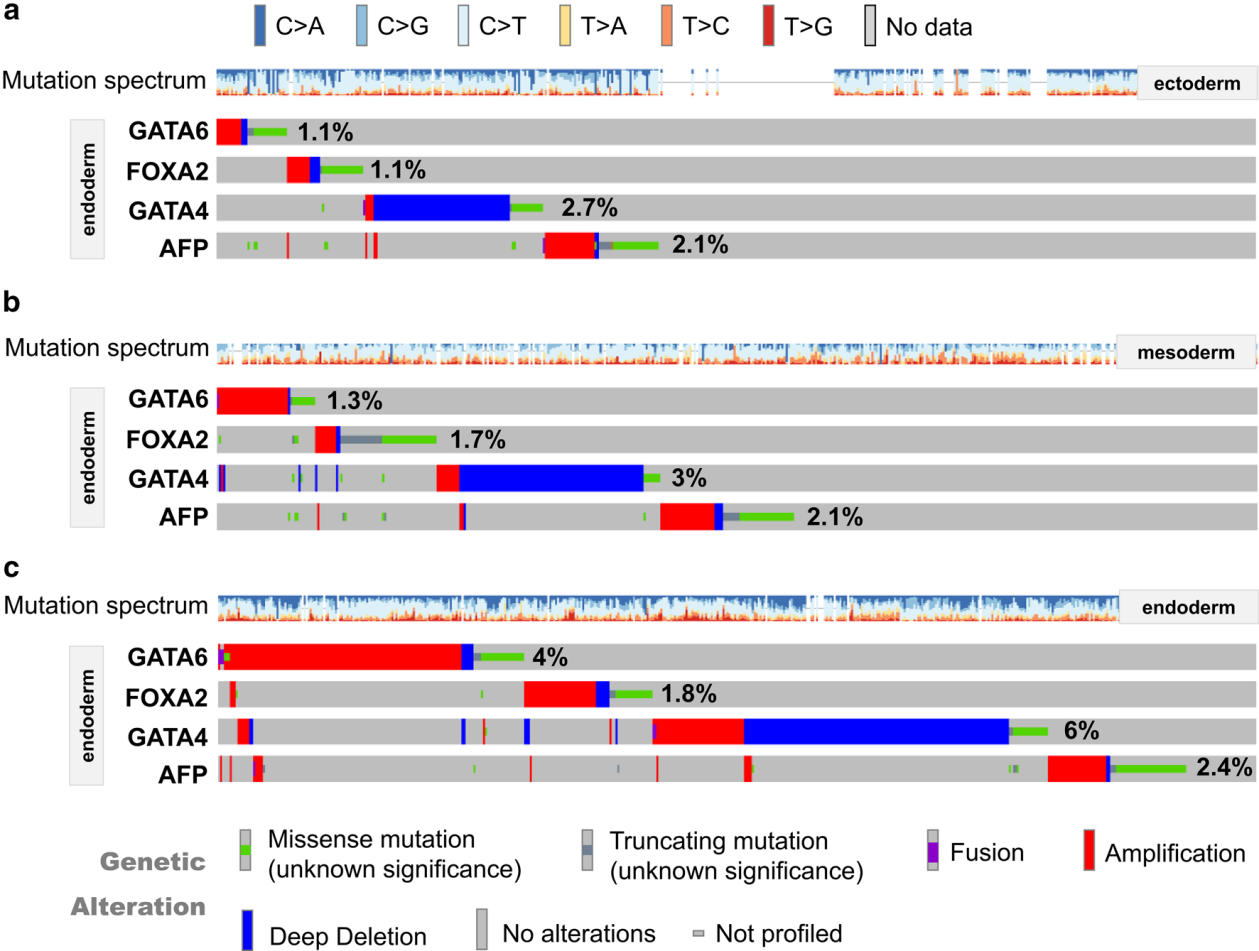
Mutation analysis of endoderm markers. **a** Ectoderm; **b** mesoderm; **c** endoderm

We further analyzed the mutation profile of the markers of the ectoderm, mesoderm, and endoderm among the three germ layer-derived tumors, respectively. As shown in Fig. 8, there was a relatively higher mutation incidence of endoderm markers (*GATA6*, *FOXA2*, *GATA4*, *AFP*) in the endoderm group, compared with the groups of ectoderm and mesoderm. Nevertheless, we did not observe similar results for the markers of mesoderm (Additional file 7: Fig. S7) and ectoderm (Additional file 8: Fig. S8). In addition, we analyzed the expression status and survival prognosis of these markers in the tumor cases of TCGA. As shown in Additional file 9: Fig. S9, we observed a high expression trend for the germ layer markers in the tumor tissues derived from the corresponding germ layer. For instance, the expression levels of the ectoderm markers (*NES*, *TUBB3*, *SOX1*, *SALL3*) or the endoderm markers (*GATA6*, *FOXA2*, *GATA4*, *AFP*) in the ectoderm or endoderm group were more likely to be higher than the other groups. Moreover, the high expression levels of ectoderm or endoderm markers were correlated with the poor prognosis of overall survival and disease-free survival (Additional file 10: Fig. S10, *P *< 0.05).

### Genetic mutation profile of four signaling pathways

**Fig. 9:**
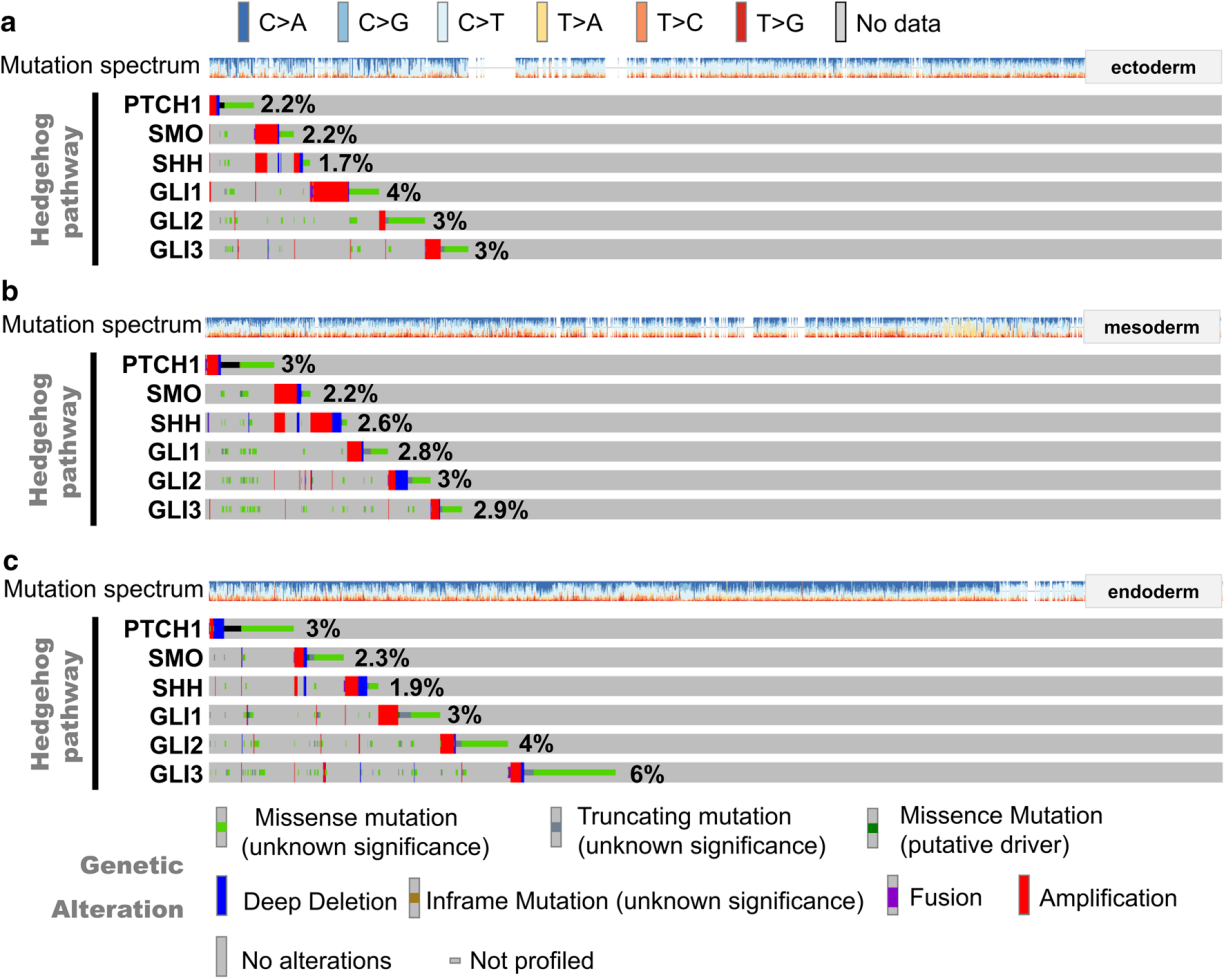
Mutation profile of Hedgehog signaling pathway. **a** Ectoderm; **b** mesoderm; **c** endoderm

Finally, we analyzed the mutation profiles of Hedgehog, Notch, TGFβ, and Wnt signaling pathways in the groups of ectoderm, mesodermal, and endoderm. As shown in Fig. 9, apart from *PTCH1* and SHH, four members (*SMO, GLI1*, *GLI2*, *GLI3*) within the Hedgehog signaling pathway genes showed a relatively higher mutation rate in the endoderm group than the other two groups. Similarly, we observed the distinct mutation rates for the selected members within the Notch (Additional file 11: Fig. S11), TGFβ (Additional file 12: Fig. S12), and Wnt (Additional file 13: Fig. S13) signaling pathways.

## Discussion

There is a spatial and temporal regulation mechanism during vertebrate embryonic development [8, 14, 30, 31]. The cells in the normal mature tissues or organs lacking the embryonic or reproductive cell-specific gene expression may suffer from the potential genetic mutation, epigenetic modification, and disordered regulatory mechanism, upon the complicated environment stimuli, which may contribute to the occurrence of benign or malignant tumors [14–16]. However, the two processes share certain similar gene regulation or signaling pathways [5, 14, 15]. The occurrence and progression of tumors may be a special way of tissue or organ development. Considering the potential association between embryonic development and carcinogenesis, we performed integration and comparative analysis regarding the genomic mutation profile of tumor cases available at the public-funded TCGA database in the groups of ectoderm, mesoderm, and endoderm.

We found that several highly frequent gene variants exhibit the different distribution characteristics and genetic mutation profiles in the ectoderm, mesoderm, and endoderm groups. For instance, the male Caucasian cases in the mesoderm group with common deletion mutations showed a better prognosis than that in the ectoderm and endoderm groups with the missense mutations. The *FGF4* gene of the Wnt pathway with the genetic alteration of “Amplification” in the ectoderm showed a higher mutation rate (10%) than other groups. Additionally, there exists the distinct mutation prevalence of the three mutation sites of the *TP53* gene, including the R175H, R273C, and R248Q, in the ectoderm, mesoderm, or endoderm-derived tumors.

The determination of three germ layers is essential for the diversification of vertebrate cells [8, 11]. Germ layer fate can be redirected by the artificial interference of several factors, such as transcription factors [13] or extracellular matrix [32]. In this study, we analyzed the mutation status of different germ layer markers in the corresponding tumors and only found that endoderm markers (*GATA6*, *FOXA2*, *GATA4*, *AFP*) in the endoderm group showed a relatively higher mutation rate than that in the other groups. However, similar results were not detected for the ectoderm markers (*NES*, *TUBB3*, *SOX1*, *SALL3*) and mesoderm markers (*MESP1*, *EOMES*, *TBXT*, *MIXL1*). The subsequent expression and prognosis analysis data showed a high expression of the four ectoderm markers in the tumor tissues in the ectoderm group, which is also correlated with the poor clinical prognosis of cases. Thus, besides the mutation mechanism, the reactivation or functional enhancement of more embryonic development-related driver genes through expression regulation is worthy of further investigation.

Our results suggested that a high proportion of children patients aged from 0 to 9 years old in the groups of ectoderm and mesoderm, but a high percentage of tumor cases aged 50–89 years old in the endoderm group. How to understand the difference in the age distribution among the three germ layer groups? In the ectoderm group, tumors were mostly in the breast, nervous system, or epidermis. Our mesoderm group includes tumors of the kidney, ovary, blood, uterus, blood, prostate, and so on. And our endoderm group mainly comprises the tumors of the bronchus, lung, colon, pancreas, thyroid, esophagus, and liver. The susceptibility of children to the ectoderm and mesoderm-derived tumors may be associated with the factor of genetic development. And the gained environmental stimuli, such as air pollution, irregular diet, smoking, or drinking, may partly contribute to the propensity of middle-aged and elderly individuals to the endoderm-derived tumors.

Although we have observed the differences in the distribution feature, mutation types, and clinical prognosis, there were no specific mutation sites with a high incidence that can effectively distinguish the tumors of different germ layers. Four members within the Hedgehog signaling pathway (*SMO*, *GLI1*, *GLI2*, *GLI3*) and four markers of endoderm (*GATA6*, *FOXA2*, *GATA4*, *AFP*) showed a relatively high mutation rate in the endoderm-derived tumors, suggesting the potential role of these driver genes or specific signaling pathways in the occurrence and development of tumors in the tissues or organs derived from endoderm. But, it should be noted that the germ layer differentiation involves the synergy of multiple driving genes or signaling pathways. We only selected a few specific genes for the preliminary analysis, based on the published evidence. The expression, mutation, and oncogenic role analysis of more driver genes involved in the development of the germ layers or the specific organs are required for a more scientific investigation. In addition, it is meaningful to integrate the clinical-pathological indicators (gender, age, tumor grade, biomarkers), disease risk factors (alcohol, virus, environment), clinical treatment (surgery, chemotherapy, radiotherapy), and other factors to establish an efficient analytical model for the investigation of the crosstalk between the embryogenesis and tumorigenesis. During this process, the influence of promiscuous genes or non-critical mutation sites should be excluded.

## Conclusions

In summary, we took advantage of TCGA datasets to explore the genetic mutation spectrum of different germ layer-derived tumors in terms of embryonic development. We failed to obtain the mutated genes or mutation sites with high frequency, which are relatively restricted to the ectoderm, mesoderm, or endoderm-derived tumors. There was a difference among the three germ layers in the issues of distribution characteristics, survival status, and mutation profile. The synergistic mutation effect of driver genes involved in the germ layer development may contribute to the functional connection between embryogenesis and tumorigenesis. Additional work is still required to further investigate the role of gene regulatory networks of germ layer specification and organogenesis in the development of specific cancers.

## Supplementary information

**Additional file 1: Fig. S1.** Schematic diagram of early embryonic development.

**Additional file 2: Fig. S2.** Classification of TCGA tumor cases.

**Additional file 3: Fig. S3.** Overall survival curve analysis by race and gender. **a** Asian race; **b** White race; **c** Black or African American; **d** male; **e** males in the white race; **f** Female.

**Additional file 4: Fig. S4.** Analysis of most mutated genes. **a** Top ten most mutated mRNAs, lincRNAs, or miRNAs in the ectoderm group; **b** Top ten most mutated mRNAs, lincRNAs, or miRNAs in the mesoderm group; **c** Top ten most mutated mRNAs, lincRNAs, or miRNAs in the endoderm group; **d** Top ten ectoderm, mesoderm, or endoderm-specific mutated genes. **e** Disease type of cases with the mutated *RP11*-*65L 19.4*, *PPP1R2P9*, *AP000345.1*, *C16orf95*, *SNORA71E* genes.

**Additional file 5: Fig. S5.** Analysis of mostly mutated gene sites. **a** An Venn diagram analysis of the three groups; **b** Top ten ectoderm-specific mutation sites; disease type of cases with the missense *NRAS* Q61K and Splice Acceptor GATA3 X309_splice mutation sites. **c** Top ten mesoderm-specific mutation sites; disease type of cases with the deletion-3 prime UTR ADAR, UTR BHLHE40, UTR ZFX. **d** Top ten endoderm-specific mutation sites; disease type of cases with the frameshift CEP350 N2889Ifs*10, 3 Prime UTR CHIC2.

**Additional file 6: Fig. S6.** Analysis of mostly mutation sites within *PIK3CA* and *TP53* genes. **a** *PIK3CA* E545K, H1047R; **b** *TP53* R175H, R248Q, R273C.

**Additional file 7: Fig. S7.** Mutation analysis of mesoderm markers. **a** ectoderm; **b** mesoderm; **c** endoderm.

**Additional file 8: Fig. S8.** Mutation analysis of ectoderm markers. **a** ectoderm; **b** mesoderm; **c** endoderm.

**Additional file 9: Fig. S9.** Expression analysis of three germ layer markers

**Additional file 10: Fig. S10.** Survival analysis of three germ layer markers. **a** OS for ectoderm; **b** DFS for ectoderm; **c** OS for mesoderm; **d** DFS for mesoderm; **e** OS for endoderm; **f** DFS for endoderm.

**Additional file 11: Fig. S11.** Mutation profile of Notch signaling pathway. **a** ectoderm; **b** mesoderm; **c** endoderm.

**Additional file 12: Fig. S12.** Mutation profile of TGFβ signaling pathway. **a** ectoderm; **b** mesoderm; **c** endoderm.

**Additional file 13: Fig. S13.** Mutation profile of Wnt signaling pathway. **a** ectoderm; **b** mesoderm; **c** endoderm.

## Abbreviations

TCGA: The cancer genome atlas
LGG: Brain lower-grade glioma
GBM: Glioblastoma multiforme
OS: Overall survival
DFS: Disease free survival

## References

[1] Gillott C, Gillott C (1980). Embryonic development. Entomology.

[2] Liu A-X, Liu X-M, Zhang Y-L, Huang H-F, Xu C-M, Huang H-F, Sheng J-Z (2014). Physiology of embryonic development. Gamete and embryo-fetal origins of adult diseases.

[3] Cook CS, Sulik KK, Wright KW, Wright KW, Spiegel PH, Thompson LS (2006). Embryology. Handbook of pediatric neuro-ophthalmology.

[4] Donkelaar HJ, Donkelaar HJ, Lammens M, Hori A (2006). Mechanisms of Development. Clinical neuroembryology: development and developmental disorders of the human central nervous system.

[5] Favarolo MB, López SL (2018). Notch signaling in the division of germ layers in bilaterian embryos. Mech Dev.

[6] Ghanavatinejad F, Fard Tabrizi ZP, Omidghaemi S, Sharifi E, Moller SG, Jami MS (2019). Protein biomarkers of neural system. J Otol..

[7] Donkelaar HJ, Yamada S, Shiota K, van der Vliet T, Donkelaar HJ, Lammens M, Hori A (2014). Overview of the development of the human brain and spinal cord. Clinical neuroembryology: development and developmental disorders of the human central nervous system.

[8] Kiecker C, Bates T, Bell E (2016). Molecular specification of germ layers in vertebrate embryos. Cell Mol Life Sci.

[9] Tseng W-C, Munisha M, Gutierrez JB, Dougan ST, Pelegri F, Danilchik M, Sutherland A (2017). Establishment of the Vertebrate Germ Layers. Vertebrate Development: Maternal to Zygotic Control.

[10] Kim PT, Ong CJ (2012). Differentiation of definitive endoderm from mouse embryonic stem cells. Results Probl Cell Differ.

[11] Fukuda K, Kikuchi Y (2005). Endoderm development in vertebrates: fate mapping, induction and regional specification. Dev Growth Differ.

[12] Lombardi J, Lombardi J (1998). Embryogenesis. Comparative vertebrate reproduction.

[13] Flickinger R (2015). AT-rich repetitive DNA sequences, transcription frequency and germ layer determination. Mech Dev.

[14] Quail DF, Siegers GM, Jewer M, Postovit LM (2013). Nodal signalling in embryogenesis and tumourigenesis. Int J Biochem Cell Biol.

[15] Fonar Y, Frank D (2011). FAK and WNT signaling: the meeting of two pathways in cancer and development. Anticancer Agents Med Chem.

[16] Spadafora C (2015). A LINE-1-encoded reverse transcriptase-dependent regulatory mechanism is active in embryogenesis and tumorigenesis. Ann N Y Acad Sci.

[17] Tomczak K, Czerwinska P, Wiznerowicz M (2015). The Cancer Genome Atlas (TCGA): an immeasurable source of knowledge. Contemp Oncol (Pozn)..

[18] Deng M, Bragelmann J, Schultze JL, Perner S (2016). Web-TCGA: an online platform for integrated analysis of molecular cancer data sets. BMC Bioinform.

[19] Tang Z, Kang B, Li C, Chen T, Zhang Z (2019). GEPIA2: an enhanced web server for large-scale expression profiling and interactive analysis. Nucleic Acids Res.

[20] Paccola Mesquita FC, Hochman-Mendez C, Morrissey J, Sampaio LC, Taylor DA (2019). Laminin as a Potent Substrate for Large-Scale Expansion of Human Induced Pluripotent Stem Cells in a Closed Cell Expansion System. Stem Cells Int..

[21] Zhang D, Wu X, Liu X, Cai C, Zeng G, Rohozinski J (2017). Piwil2-transfected human fibroblasts are cancer stem cell-like and genetically unstable. Oncotarget..

[22] Guo NN, Liu LP, Zhang YX, Cai YT, Guo Y, Zheng YW (2019). Early prediction of the differentiation potential during the formation of human iPSC-derived embryoid bodies. Biochem Biophys Res Commun.

[23] Liu H, Zhang S, Zhao L, Zhang Y, Li Q, Chai X (2016). Resveratrol Enhances Cardiomyocyte Differentiation of Human Induced Pluripotent Stem Cells through Inhibiting Canonical WNT Signal Pathway and Enhancing Serum Response Factor-miR-1 Axis. Stem Cells Int..

[24] Diomede F, Zini N, Pizzicannella J, Merciaro I, Pizzicannella G, D’Orazio M (2018). 5-Aza Exposure Improves Reprogramming Process Through Embryoid Body Formation in Human Gingival Stem Cells. Front Genet..

[25] Fang F, Li Z, Zhao Q, Xiong C, Ni K (2020). Analysis of multi-lineage gene expression dynamics during primordial germ cell induction from human induced pluripotent stem cells. Stem Cell Res Ther..

[26] Gao J, Aksoy BA, Dogrusoz U, Dresdner G, Gross B, Sumer SO (2013). Integrative analysis of complex cancer genomics and clinical profiles using the cBioPortal. Sci Signal..

[27] Cerami E, Gao J, Dogrusoz U, Gross BE, Sumer SO, Aksoy BA (2012). The cBio cancer genomics portal: an open platform for exploring multidimensional cancer genomics data. Cancer Discov.

[28] Pelullo M, Zema S, Nardozza F, Checquolo S, Screpanti I, Bellavia D (2019). Wnt, Notch, and TGF-β pathways impinge on hedgehog signaling complexity: an open window on cancer. Front Genet..

[29] Pucéat M (2007). TGFbeta in the differentiation of embryonic stem cells. Cardiovasc Res.

[30] Takemoto T (2013). Mechanism of cell fate choice between neural and mesodermal development during early embryogenesis. Congenit Anom (Kyoto)..

[31] Reik W, Dean W, Walter J (2001). Epigenetic reprogramming in mammalian development. Science.

[32] Williams ML, Bhatia SK (2014). Engineering the extracellular matrix for clinical applications: endoderm, mesoderm, and ectoderm. Biotechnol J.

