# Supplementary figures and images for: Differential genetic mutations of ectoderm, mesoderm, and endoderm-derived tumors in TCGA database
Source: Cancer Cell Int. 2020 Dec 11;20:595. doi: 10.1186/s12935-020-01678-x (PMC7730784; doi:10.1186/s12935-020-01678-x)

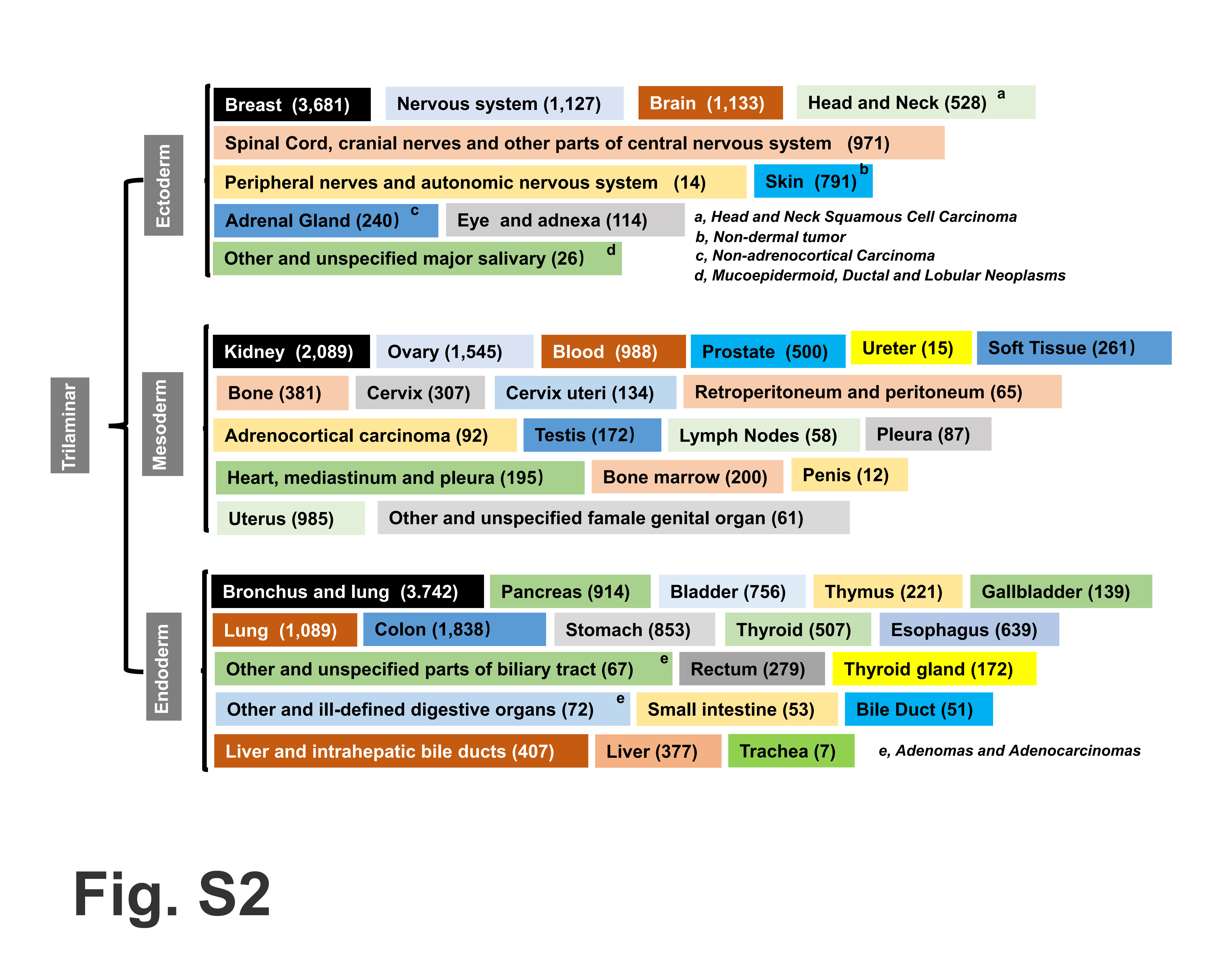

Supplement: Supplementary file 2 — Additional file 2: Fig. S2. Classification of TCGA tumor cases. [file 12935_2020_1678_MOESM2_ESM.tif]

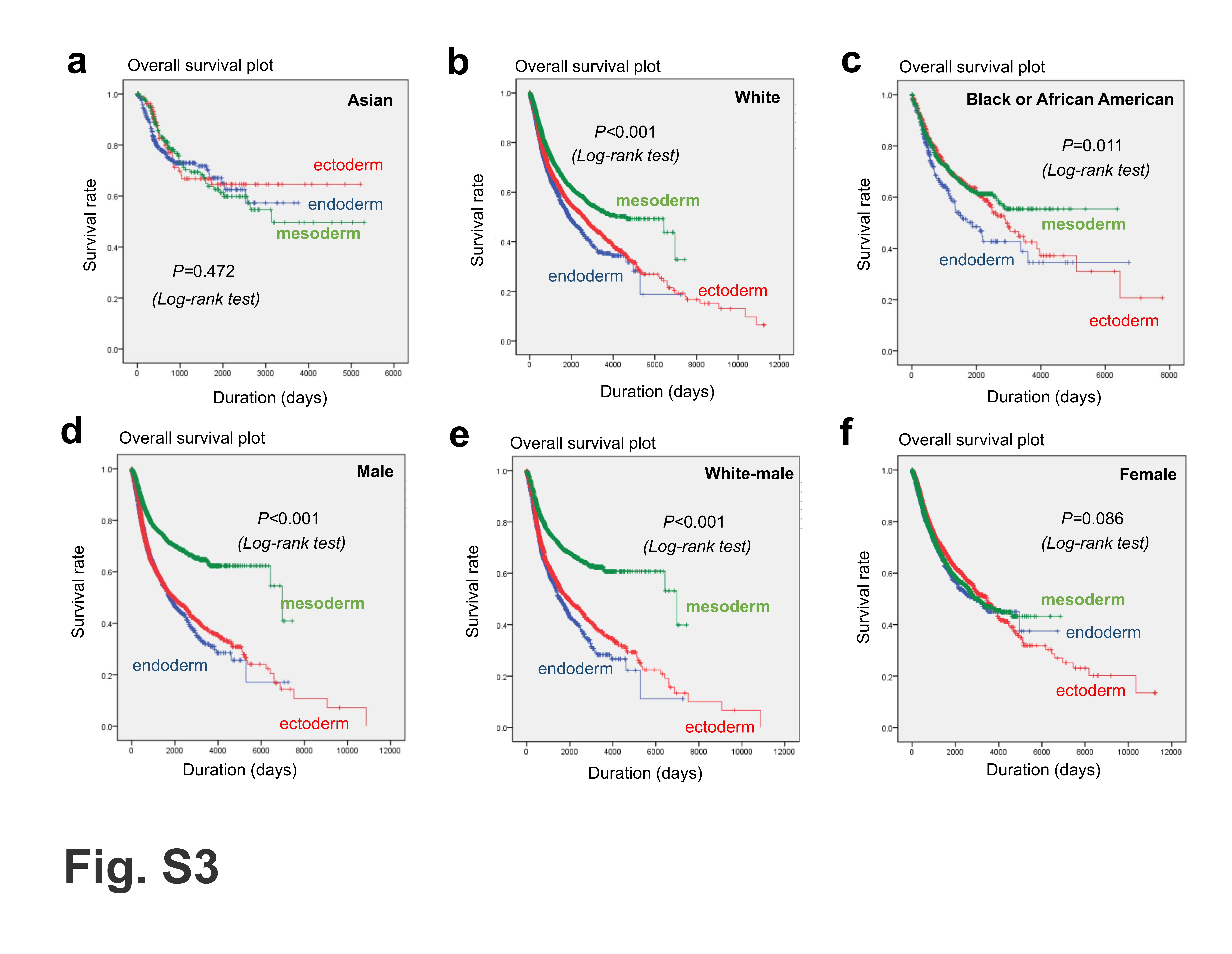

Supplement: Supplementary file 3 — Additional file 3: Fig. S3. Overall survival curve analysis by race and gender. a Asian race; b White race; c Black or African American; d male; e males in the white race; f Female. [file 12935_2020_1678_MOESM3_ESM.tif]

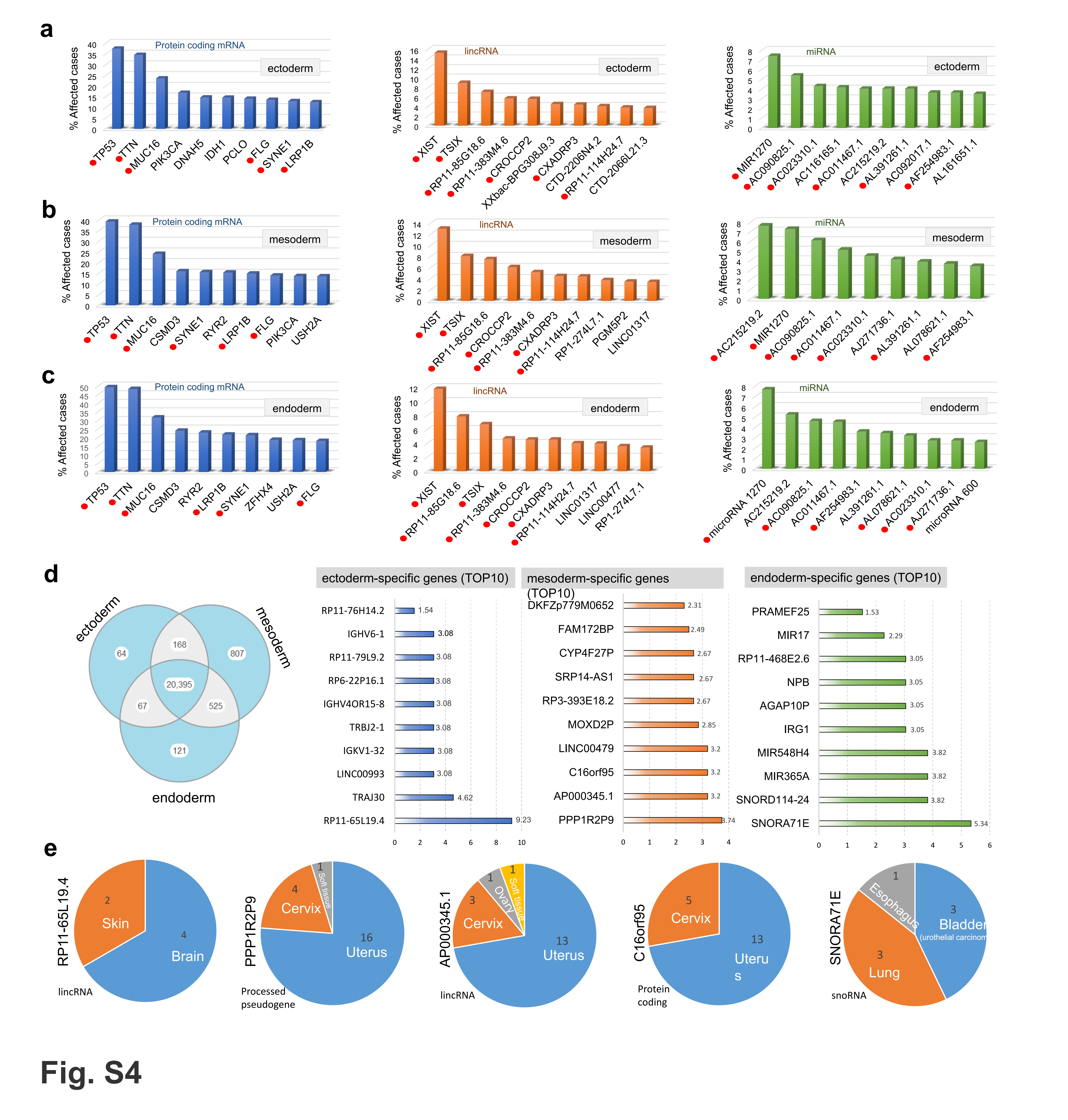

Supplement: Supplementary file 4 — Additional file 4: Fig. S4. Analysis of most mutated genes. a Top ten most mutated mRNAs, lincRNAs, or miRNAs in the ectoderm group; b Top ten most mutated mRNAs, lincRNAs, or miRNAs in the mesoderm group; c Top ten most mutated mRNAs, lincRNAs, or miRNAs in the endoderm group; d Top ten ectoderm, mesoderm, or endoderm-specific mutated genes. e Disease type of cases with the mutated RP11-65L 19.4, PPP1R2P9, AP000345.1, C16orf95, SNORA71E genes. [file 12935_2020_1678_MOESM4_ESM.tif]

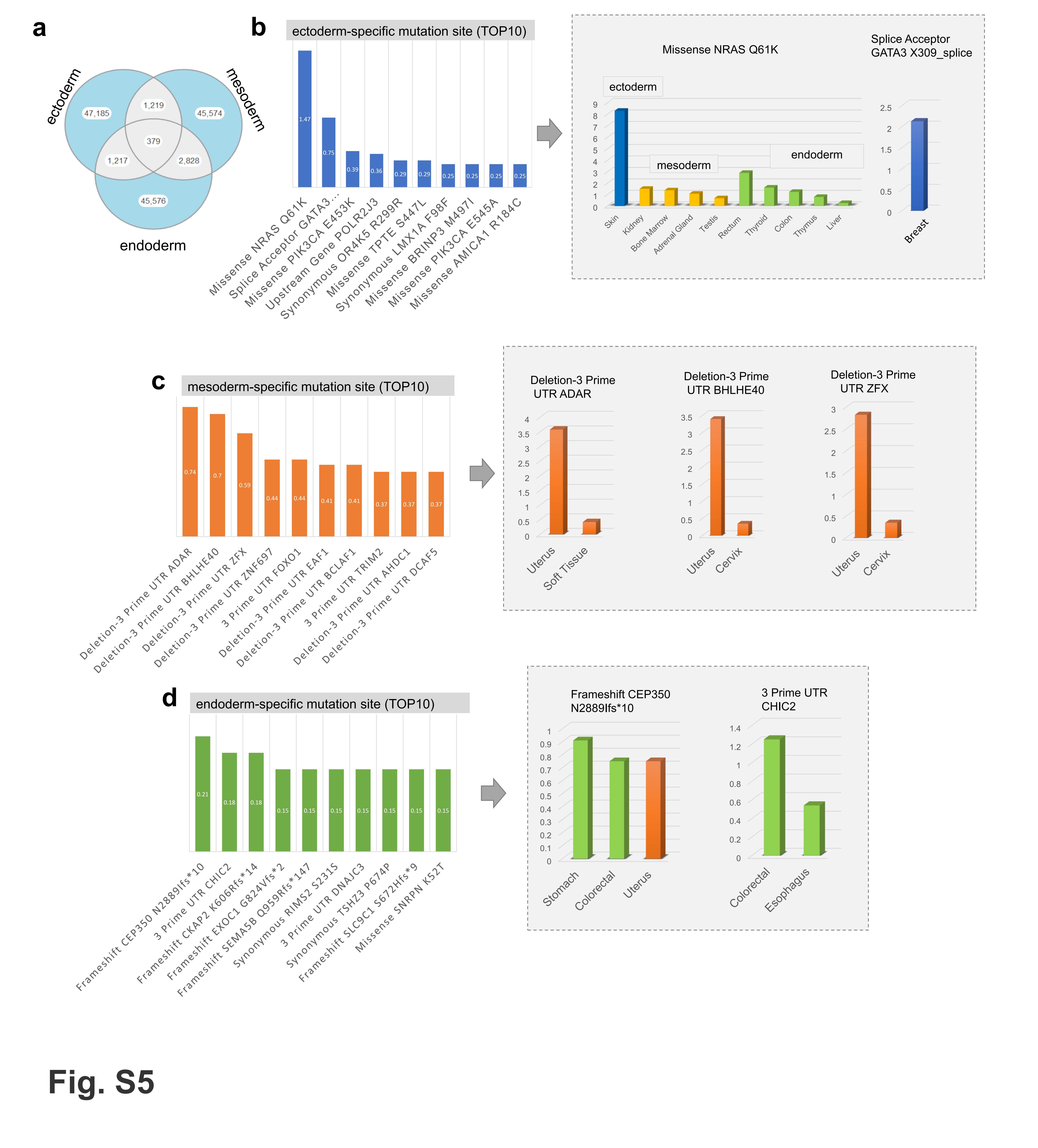

Supplement: Supplementary file 5 — Additional file 5: Fig. S5. Analysis of mostly mutated gene sites. a An Venn diagram analysis of the three groups; b Top ten ectoderm-specific mutation sites; disease type of cases with the missense NRAS Q61K and Splice Acceptor GATA3 X309_splice mutation sites. c Top ten mesoderm-specific mutation sites; disease type of cases with the deletion-3 prime UTR ADAR, UTR BHLHE40, UTR ZFX. d Top ten endoderm-specific mutation sites; disease type of cases with the frameshift CEP350 N2889Ifs*10, 3 Prime UTR CHIC2. [file 12935_2020_1678_MOESM5_ESM.tif]

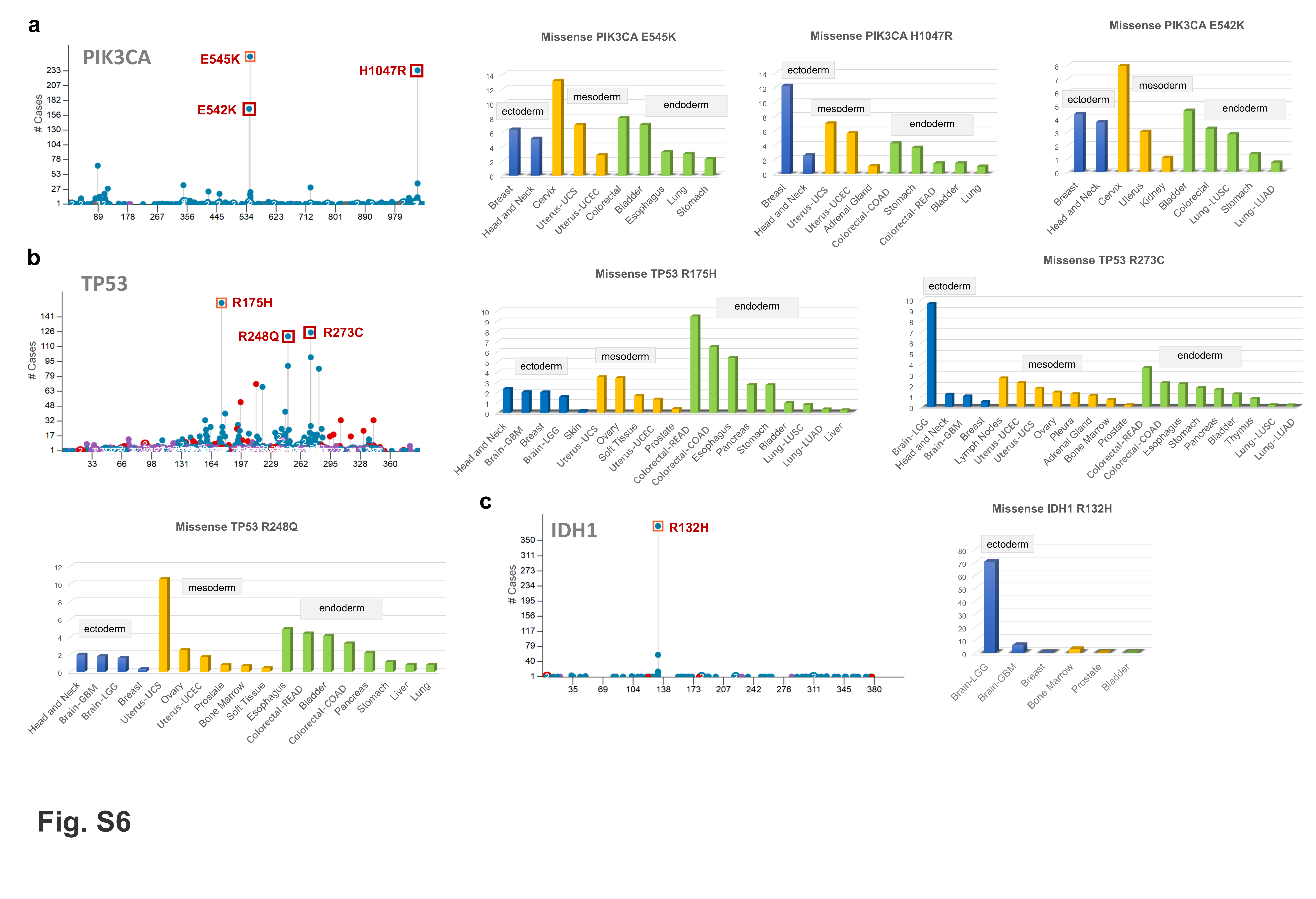

Supplement: Supplementary file 6 — Additional file 6: Fig. S6. Analysis of mostly mutation sites within PIK3CA and TP53 genes. a PIK3CA E545K, H1047R; b TP53 R175H, R248Q, R273C. [file 12935_2020_1678_MOESM6_ESM.tif]

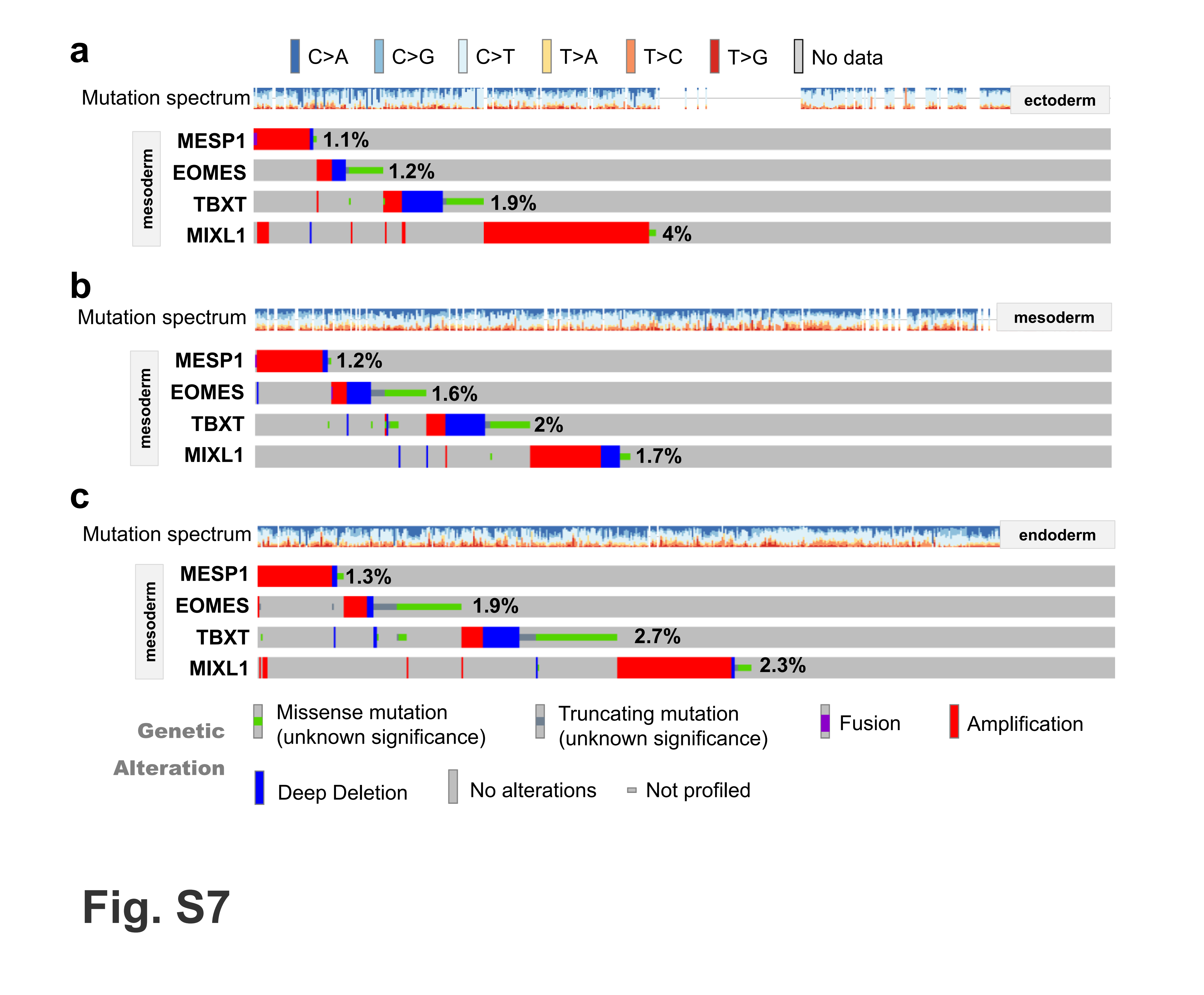

Supplement: Supplementary file 7 — Additional file 7: Fig. S7. Mutation analysis of mesoderm markers. a ectoderm; b mesoderm; c endoderm. [file 12935_2020_1678_MOESM7_ESM.tif]

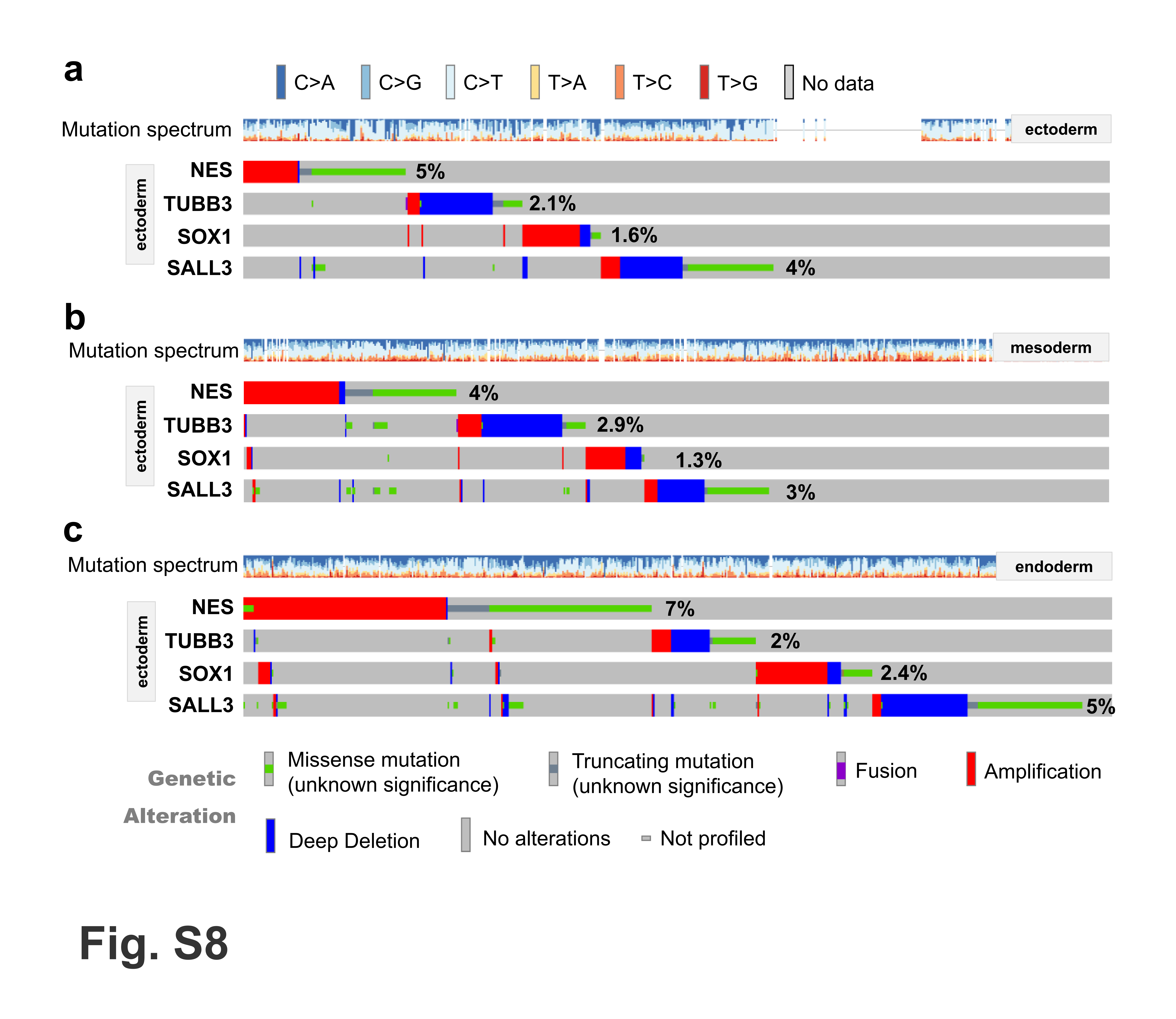

Supplement: Supplementary file 8 — Additional file 8: Fig. S8. Mutation analysis of ectoderm markers. a ectoderm; b mesoderm; c endoderm. [file 12935_2020_1678_MOESM8_ESM.tif]

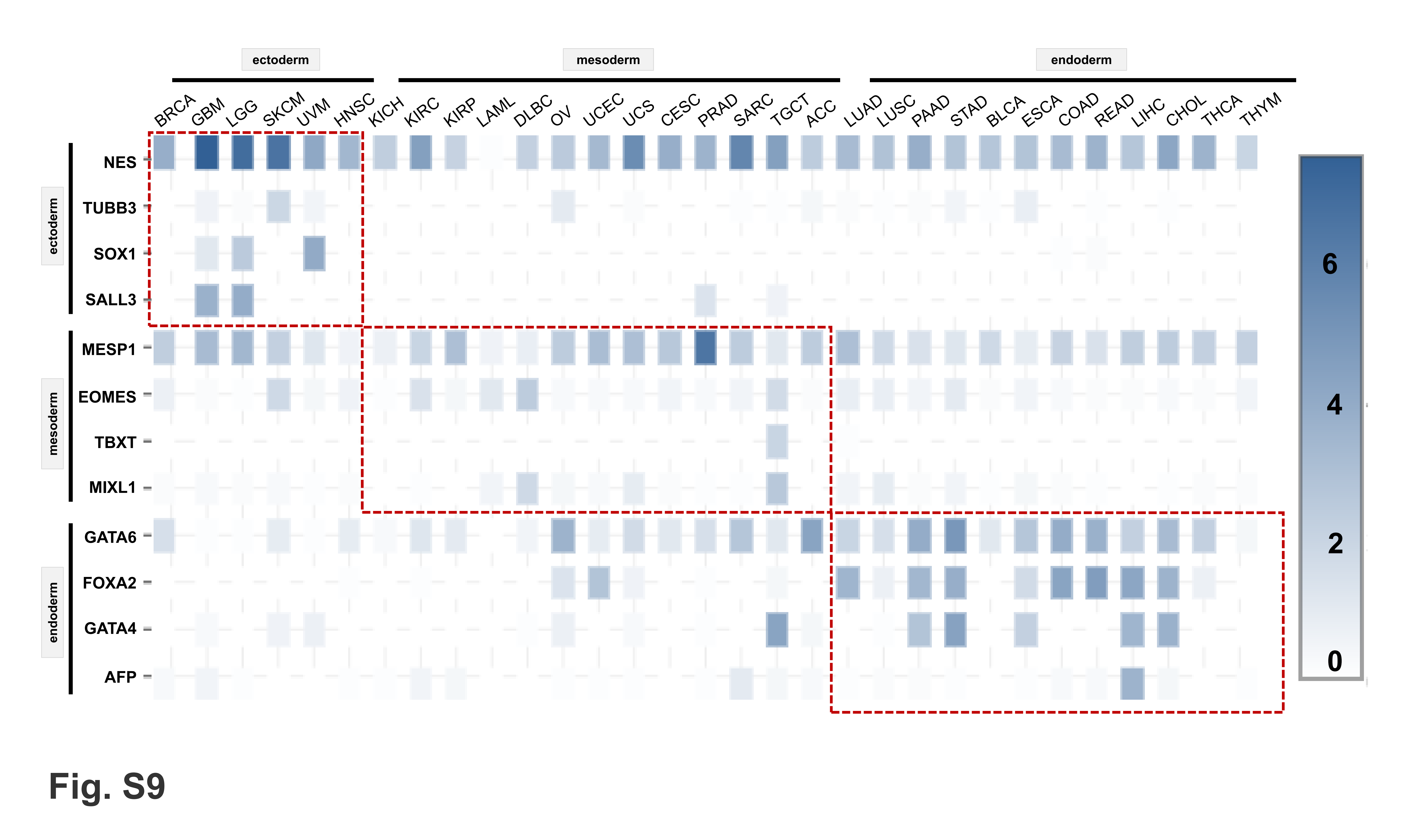

Supplement: Supplementary file 9 — Additional file 9: Fig. S9. Expression analysis of three germ layer markers [file 12935_2020_1678_MOESM9_ESM.tif]

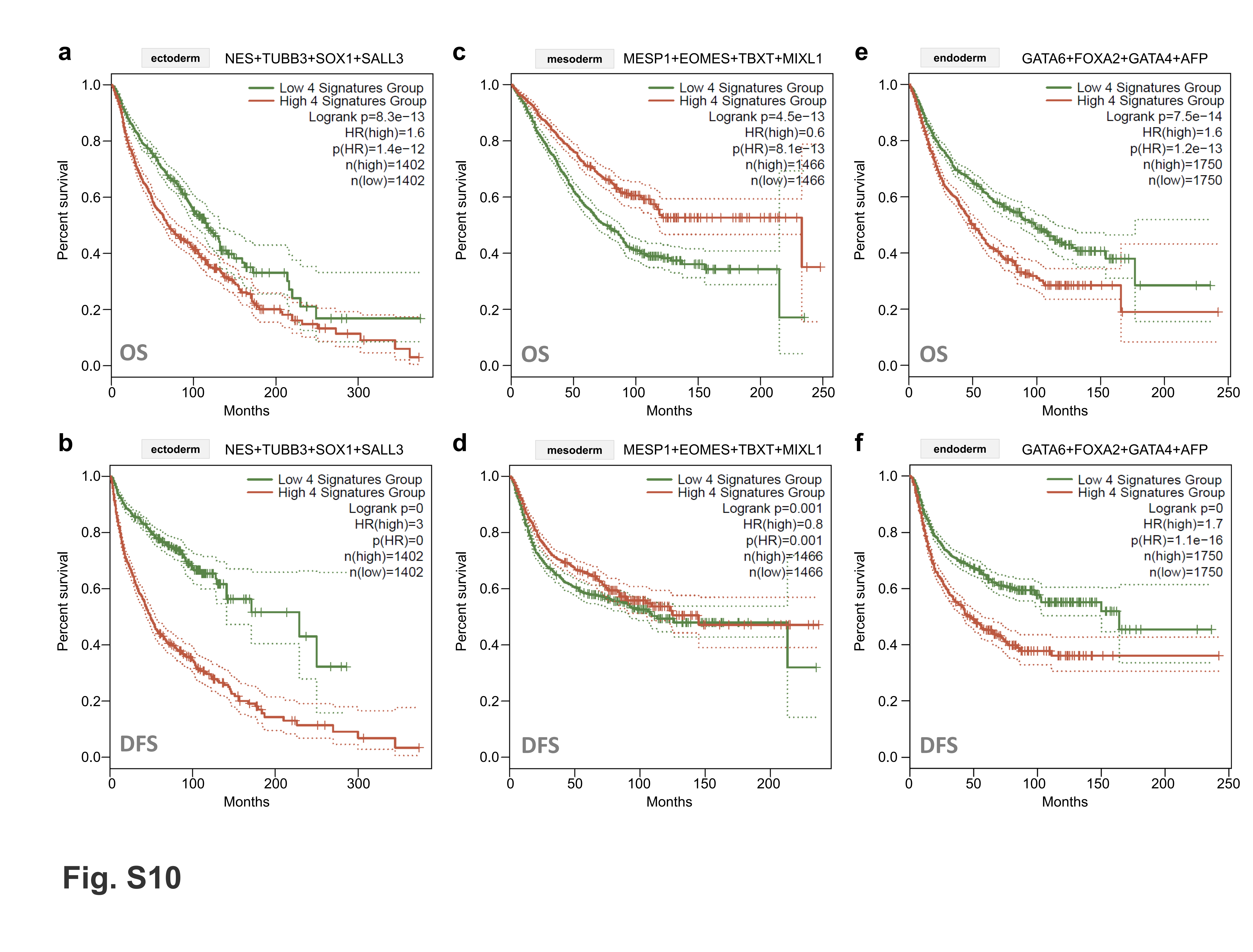

Supplement: Supplementary file 10 — Additional file 10: Fig. S10. Survival analysis of three germ layer markers. a OS for ectoderm; b DFS for ectoderm; c OS for mesoderm; d DFS for mesoderm; e OS for endoderm; f DFS for endoderm. [file 12935_2020_1678_MOESM10_ESM.tif]

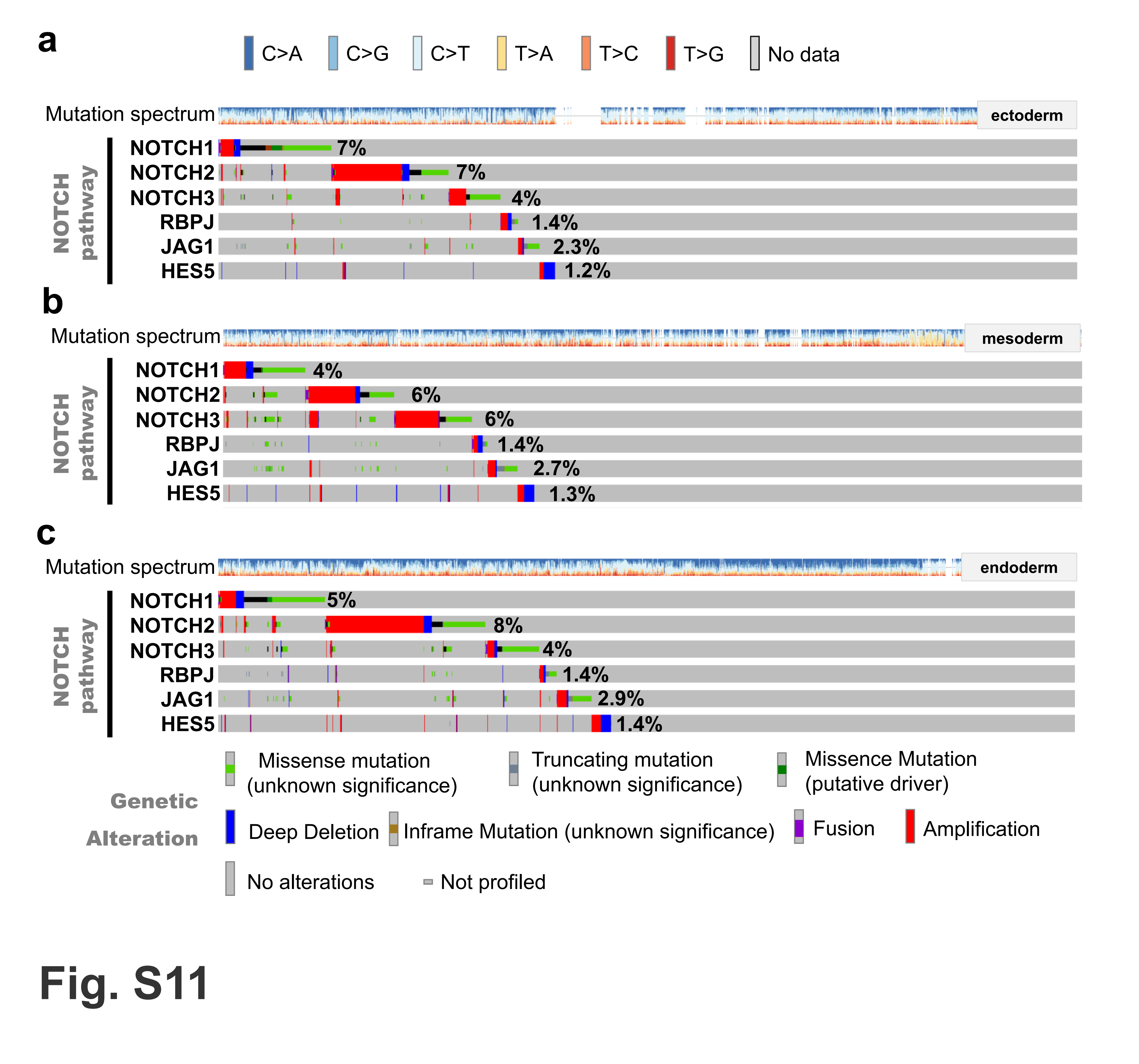

Supplement: Supplementary file 11 — Additional file 11: Fig. S11. Mutation profile of Notch signaling pathway. a ectoderm; b mesoderm; c endoderm. [file 12935_2020_1678_MOESM11_ESM.tif]

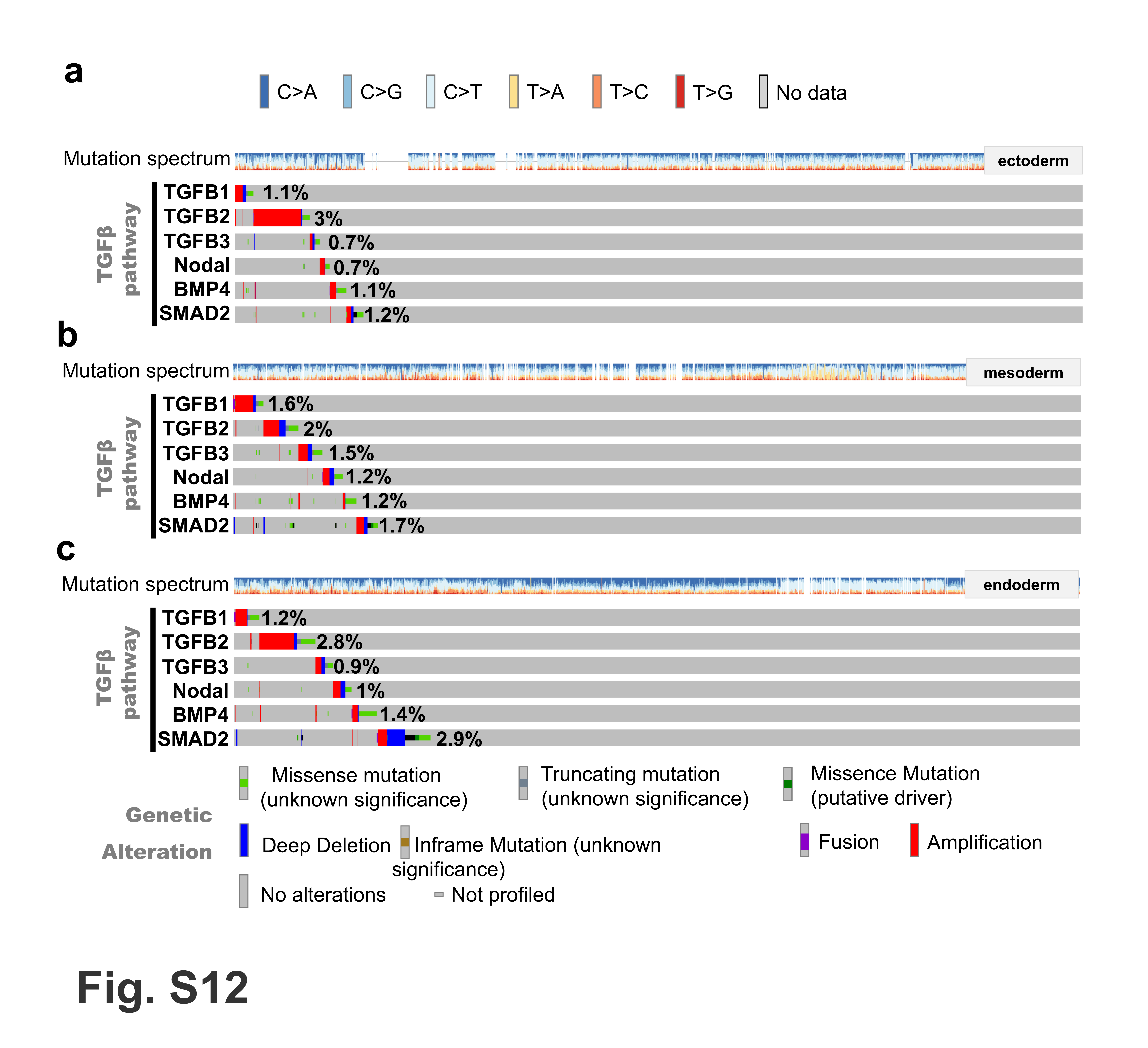

Supplement: Supplementary file 12 — Additional file 12: Fig. S12. Mutation profile of TGFβ signaling pathway. a ectoderm; b mesoderm; c endoderm. [file 12935_2020_1678_MOESM12_ESM.tif]

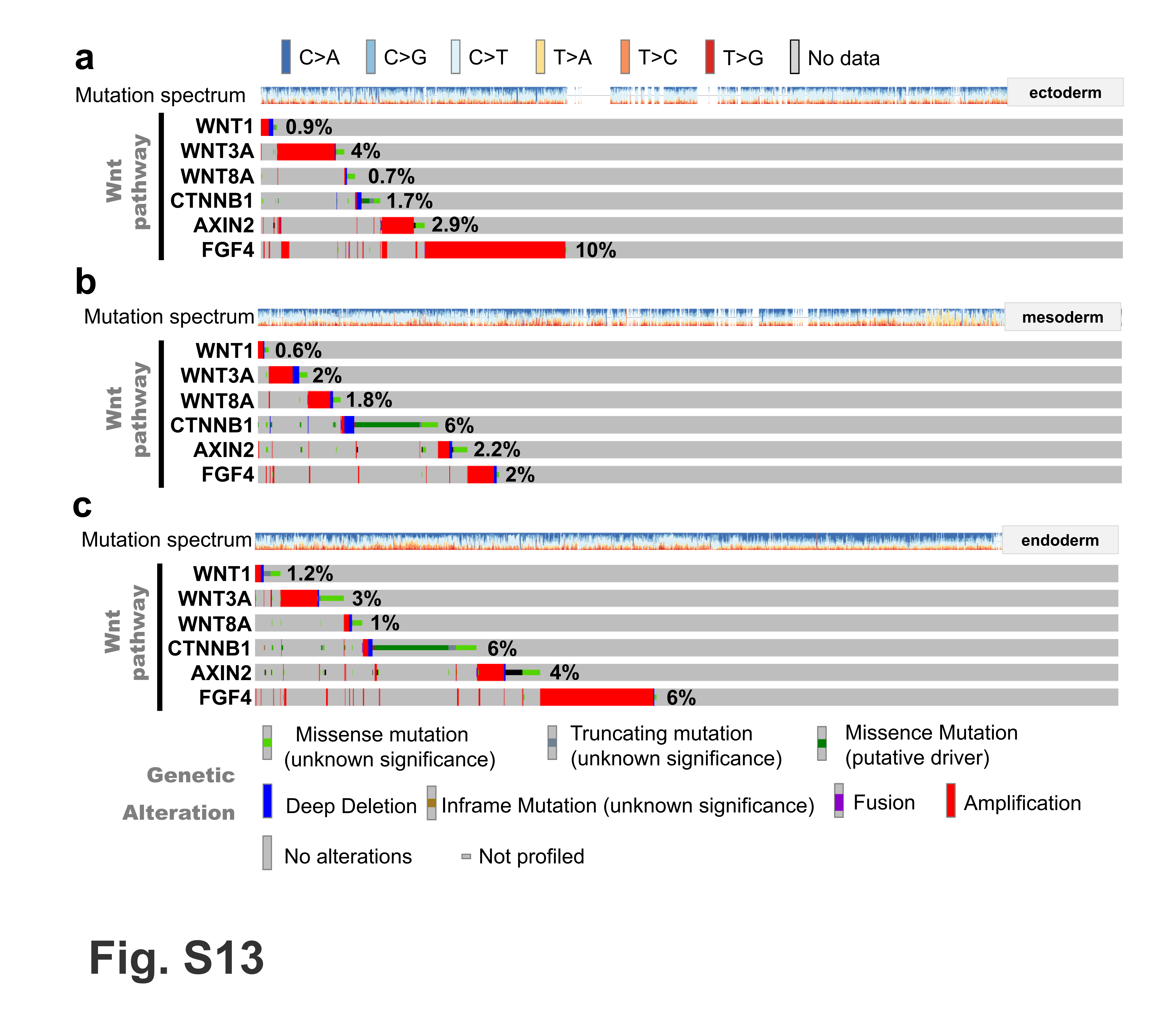

Supplement: Supplementary file 13 — Additional file 13: Fig. S13. Mutation profile of Wnt signaling pathway. a ectoderm; b mesoderm; c endoderm. [file 12935_2020_1678_MOESM13_ESM.tif]
